# Streptopyridines, volatile pyridine alkaloids produced by *Streptomyces* sp. FORM5

**DOI:** 10.3762/bjoc.10.146

**Published:** 2014-06-24

**Authors:** Ulrike Groenhagen, Michael Maczka, Jeroen S Dickschat, Stefan Schulz

**Affiliations:** 1 Institut für Organische Chemie, Technische Universität Braunschweig, Hagenring 30, 38106 Braunschweig, Germany

**Keywords:** headspace analysis, natural products, polyketide biosynthesis, pyridine derivatives, streptazolin, volatile compounds

## Abstract

*Streptomyces* sp. FORM5 is a bacterium that is known to produce the antibiotic streptazolin and related compounds. We investigated the strain for the production of volatiles using the CLSA (closed-loop stripping analysis) method. Liquid and agar plate cultures revealed the formation of new 2-alkylpyridines (streptopyridines), structurally closely related to the already known 2-pentadienylpiperidines. The structures of the streptopyridines A to E were confirmed by total synthesis. The analysis of the liquid phase by solvent extraction or extraction with an Oasis adsorbent showed that streptazolin and 2-pentadienylpiperidine are the major compounds, while the streptopyridines are only minor components. In the gas phase, only the streptopyridines could be detected. Therefore, an orthogonal set of analysis is needed to assess the metabolic profile of bacteria, because volatile compounds are obviously overlooked by traditional analytical methods. The streptopyridines are strain specific volatiles that are accompanied by a broad range of headspace constituents that occur in many actinomycetes. Volatiles might be of ecological importance for the producing organism, and, as biosynthetic intermediates or shunt products, they can be useful as indicators of antibiotic production in a bacterium.

## Introduction

Actinomycetes are excellent producers of diverse and bioactive secondary metabolites. These metabolites belong to many different structural classes including polyketides, nonribosomal peptides, terpenoids, alkaloids, lipids and others. Such compounds became a major source of biologically active natural products as antibiotics, cytotoxic compounds, immunosuppressants etc. In addition, actinomycetes are also able to produce and release a wide variety of volatile compounds with bouquets composed of up to 100 different compounds [1–6]. Major volatile classes comprise aliphatic compounds derived from fatty acid metabolism, terpenes, aromatic compounds, sulfur compounds, and pyrazines [1]. Apart from pyrazines, indole, and a few strain specific compounds [2] such as methyl pyrrole-2-carboxylate, emitted by *Stackebrandtia nassauensis*, or 2-acetylpyrrole from *Saccharopolyspora erythraea*, volatile alkaloids are rarely produced by actinomycetes.

**Figure 1:**
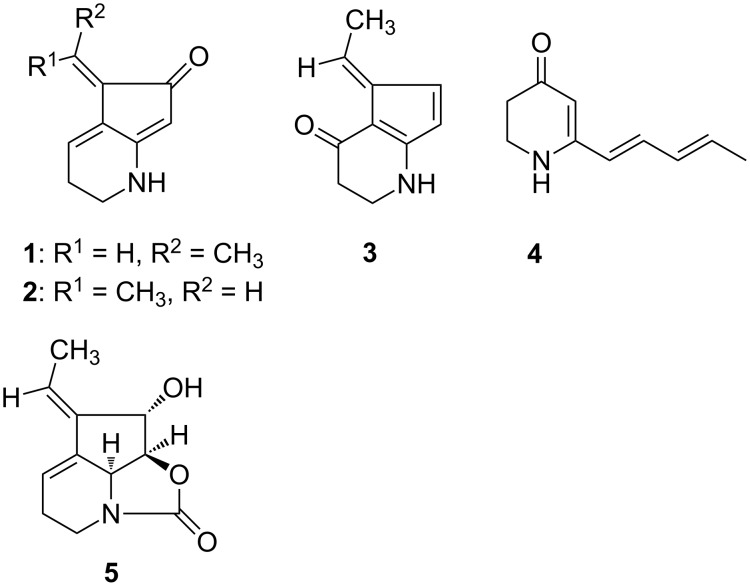
Alkaloids produced by *Streptomyces* strain FORM5.

We became interested in the strain *Streptomyces* sp. FORM5 to elucidate whether volatile formation is linked to the production of other, usually less volatile secondary metabolites and whether different compounds can be detected by headspace analysis [7] compared to commonly used solvent extraction or adsorption/extraction procedures. Strain FORM5 has been reported to produce the tetrahydrocyclopenta[*b*]pyridine derivatives streptazones B_1_ (**1**), B_2_ (**2**), C (**3**), the 4-pyridone derivative streptazone D (**4**) with a pentadienyl side chain, and streptazolin (**5**) (Figure 1) [8]. Streptazolin is produced by several streptomycetes [8–12], and is formed biosynthetically by a polyketide mechanism [13]. The respective polyketide synthase gene cluster has not been identified yet.

The streptazones are relatively small compounds suggesting that they may be volatile enough to find them in the headspace above bacterial cultures, although the presence of hydrogen bond donor and acceptor sites hints to good solubility in the aqueous phase.

In our study the volatile bouquet of the actinomycete *Streptomyces* sp. FORM5 was investigated and several new 2-alkylated pyridines were identified using the closed-loop stripping analysis (CLSA) [7] headspace technique followed by GC–MS analysis and synthesis of the target compounds for structure verification. Agar plate cultures and liquid cultures were investigated and the liquid phase analyzed for the presence of secondary metabolites. The results showed that by headspace analysis new secondary metabolites can be found that eluded earlier analysis. The separate analysis of headspace and liquid phase is complementary and in combination allows better evaluation of the metabolic potential of an investigated microorganism.

## Results and Discussion

**Figure 2:**
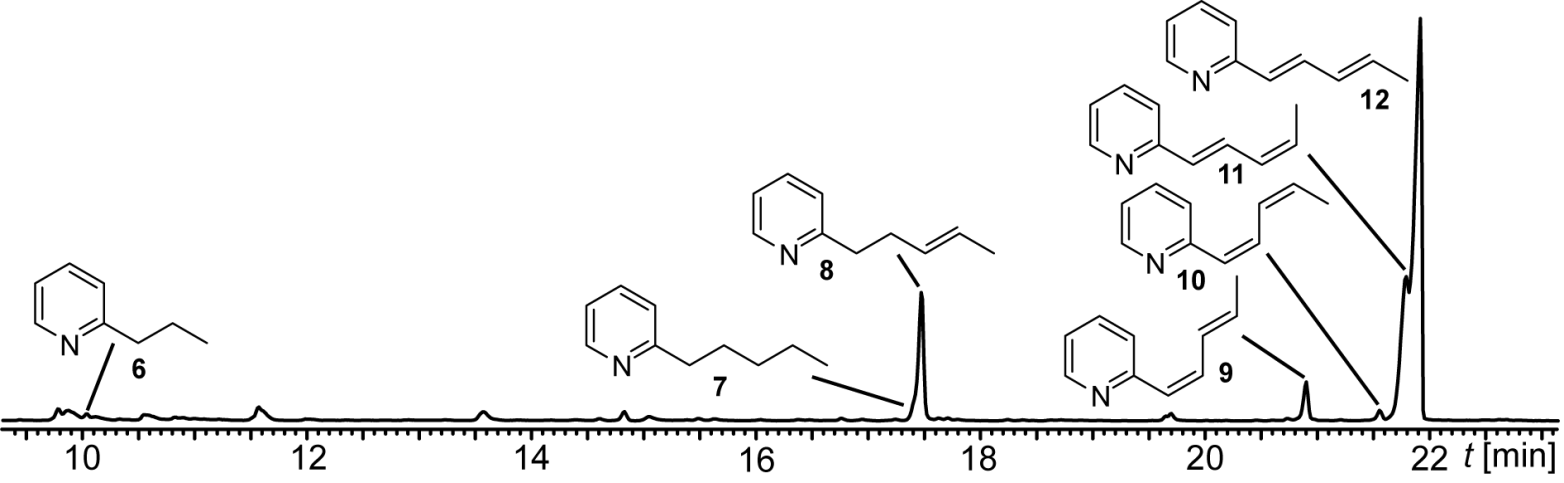
Part of the total ion chromatogram of the headspace extract of *Streptomyces* sp. FORM5 with the structures of 2-propylpyridine (**6**), 2-pentylpyridine (**7**), (*E*)-2-(pent-3-en-1-yl)pyridine (**8**), 2-((1*Z*,3*E*)-penta-1,3-dien-1-yl)pyridine (**9**), 2-((1*Z*,3*Z*)-penta-1,3-dien-1-yl)pyridine (**10**), 2-((1*E*,3*Z*)-penta-1,3-dien-1-yl)pyridine (**11**), 2-((1*E*,3*E*)-penta-1,3-dien-1-yl)pyridine (**12**).

**Figure 3:**
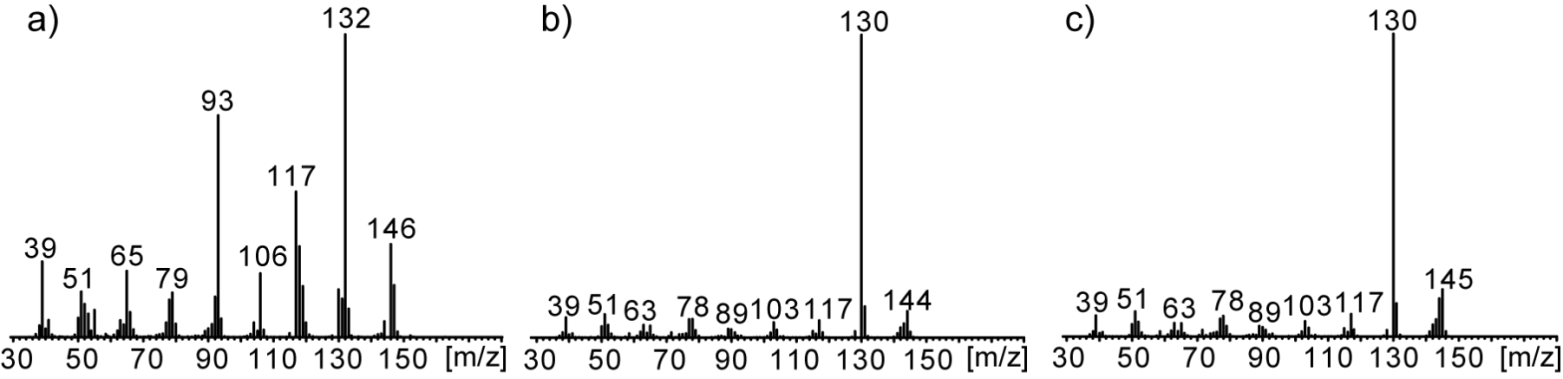
Mass spectra of a) (*E*)-2-(pent-3-en-1-yl)pyridine (streptopyridine E, **8**), b) (1*Z*,3*E*)-penta-1,3-dien-1-yl)pyridine (streptopyridine A, **9**), c) 2-((1*E*,3*E*)-penta-1,3-dien-1-yl)pyridine (streptopyridine B, **12**).

The volatiles released by agar plate cultures of strain *Streptomyces* sp. FORM5 were collected by CLSA for one day on a charcoal filter and eluted with dichloromethane. The extract was then analyzed using GC–MS. More than 40 different compounds were identified in the headspace extract (Table 1, Figure 2, and Figure S1 in Supporting Information File 1). Nevertheless, the major compounds (compounds **8**, **9**, **11**, and **12** in Figure 2) attracted our interest, because they were unknown, thus giving room for the discovery of new volatile secondary metabolites. High resolution GC–MS revealed a molecular composition of C_10_H_11_N (found 145.09136, calcd 145.08910) for the major compound **12** and C_10_H_13_N (found 147.10428, calcd 147.10475) for the minor component **8**. The mass spectrum (Figure 3) of the major compound pointed to the presence of an extended aromatic and conjugated π-system and the formation of a stable [M − 1]^+^ ion. The methyl loss to form the base peak [M − 15]^+^ indicated the presence of a methyl group in the compound. Careful analysis of the extract revealed that a small peak (**7**) eluted slightly earlier than compound **8**. The mass spectrum of compound **7** matched that of 2-pentylpyridine, present in public databases [14]. From these data we concluded that the unknown compound **12** might be a 2-(pentadienyl)pyridine with conjugated side chain and that the compounds **9**–**12** might be diastereomers. Compound **8** should carry a pentenyl side chain, while compound **6** showed a mass spectrum identical to the known one of 2-propylpyridine.

**Scheme 1:**
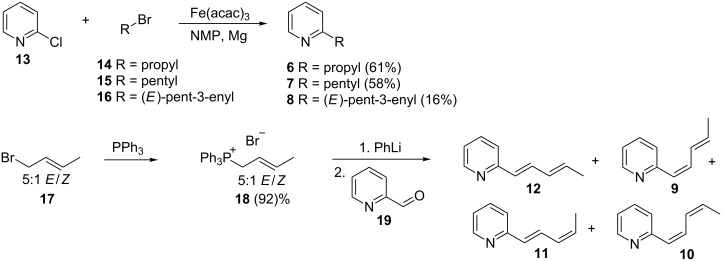
Synthesis of streptopyridines A to E (**8**–**12**) and the 2-alkylpyridines **6** and **7**.

**Figure 4:**
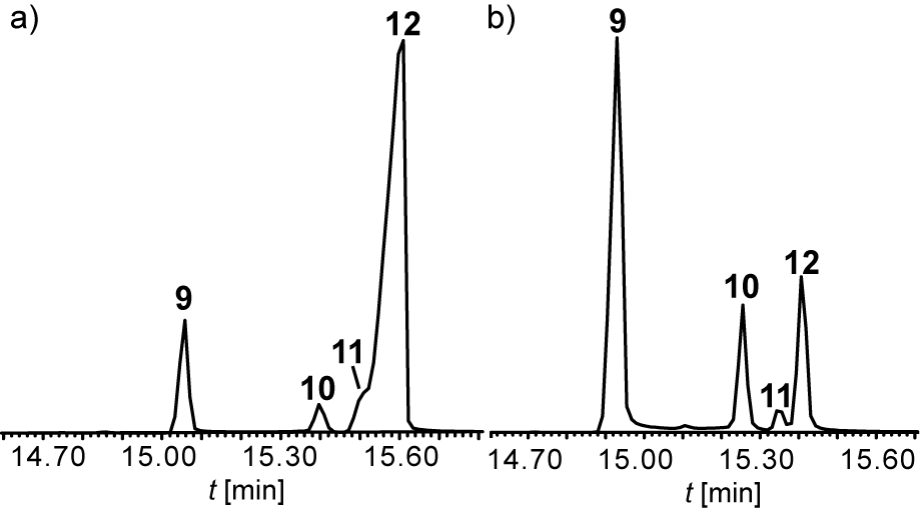
Total ion chromatograms of the product mixtures of isomers **9–12** synthesized under *E*-selective (a) and *Z*-selective Wittig reaction conditions (b).

The target compounds were then synthesized to prove the structural proposal. Pentadienylpyridines **9**–**12** were synthesized by Wittig reaction using different conditions (Scheme 1). A Wittig–Schlosser reaction [15] starting from a commercially available 5:1 *E*/*Z-*mixture of 1-bromobut-2-ene (**17**) led to preferentially (1*E*)-configured products. After conversion of **17** into the respective Wittig salt **18** and reaction with 2-pyridinecarbaldehyde (**19**) a mixture of four diastereoisomers of 2-(1,3-pentadienyl)pyridine was formed (Figure 4), all showing similar mass spectra. These four diastereomers proved to be identical to the natural compounds **9** to **12** by comparison of mass spectra and GC retention. The major compound, 2-((1*E*,3*E*)-penta-1,3-dienyl)pyridine (**12**), and the 1*Z*,3*E*-isomer **9** could be isolated in almost pure form, but a large amount of material was lost during the purification process (17% yield for compound **12** and 3% yield for compound **9** after purification). The large coupling constants ^3^*J* around 15 Hz between H-1 and H-2 (15.8 Hz) as well as H-3 and H-4 (15.1 Hz) in the side chain indicated the *E*-configuration of both double bonds for compound **12**. The mass spectrum and retention time proved to be identical to compound **12**, the major compound of the natural extract. We propose the name streptopyridine A for this new natural compound. The minor product isolated pure under these conditions was 2-((1*Z*,3*E*)-penta-1,3-dienyl)pyridine (**9**, streptopyridine B), indicated by the coupling constants ^3^*J*_1,2_ = 11.8 Hz and ^3^*J*_3,4_ = 15.1 Hz.

To delineate the stereochemistry of the other two isomers **10** and **11**, the conditions of the Wittig reaction were changed to favor *Z*-configured products by using NaHMDS as base. Again, all four diastereomers were formed (Figure 4). Together with knowledge of the structures of **9** and **12**, the product ratio in the *Z*-selective reaction indicates that compound **10** (streptopyridine C) is 2-((1*Z*,3*Z*)-penta-1,3-dienyl)pyridine, while compound **11** is 2-((1*E*,3*Z*)-penta-1,3-dienyl)pyridine (streptopyridine D): The formation of only small amounts of **11** indicates that this compound is 1*E*,3*Z*-configured, because it is disfavored by the 1*Z*-selective reaction conditions and the minor amounts of the *Z*-configured isomer in the diastereomeric mixture of Wittig salts. Conclusively, **10** must be the last possible 1*Z*,3*Z*-stereoisomer. Both compounds **10** and **11** could not be isolated in pure form, but their mass spectra and retention times were identical to those of the natural products.

The mass spectrum of compound **8** indicated that the side chain contained only one double bond, the position of which had to be determined. A 1-pentenyl side chain seemed unlikely because of the high abundance of the *m*/*z* 93 ion in the mass spectrum of **8**, which we assumed is a McLafferty ion that is commonly observed in 2-alkylpyridines [16]. Basing on the structure of streptopyridine A, a 3-pentenyl side chain seemed to be most likely. Coupling of (*E*)-3-pentenylmagnesium bromide with 2-chloropyridine under Fürstner conditions with iron(III) acetylacetonate as catalyst [17] yielded (*E*)-2-(pent-3-enyl)pyridine (**8**) that proved to be identical to the natural compound, now called streptopyridine E. The mass spectra of 2-(pent-1-enyl)pyridine and 2-(pent-2-enyl)pyridine, compounds synthesized for comparison, are shown in Supporting Information File 1 and differ from that of **8**. Finally, pyridines **6** and **7** were synthesized also by Fürstner cross-coupling and proved to be identical to the natural products.

2-Alkylpyridines have been reported earlier as aroma components, like 2-butylpyridine or 2-pentylpyridine (**7**) identified in fried chicken [18], or 2-propylpyridine (**6**), present in sesame seed oil [19], but are not known from bacteria. Natural products of bacteria containing a pyridine ring are rare. As an example, 1-(2-pyridinyl)ethanone was identified as a volatile of *Enterobacter agglomerans* [20]. Highly substituted pyridine derivatives can be found in bacterial thiopeptide antibiotics [21].

**Figure 5:**
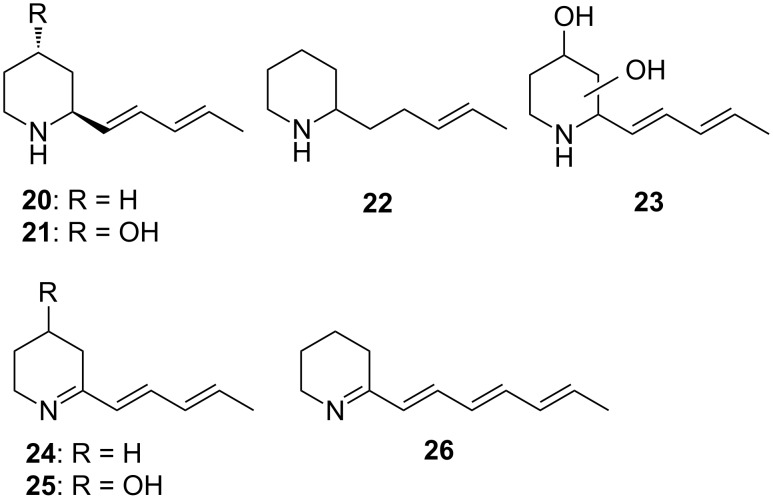
Structures of piperidine derivatives **20**–**26**.

The streptopyridines of *Streptomyces* sp. FORM5 are structurally related to known secondary metabolites by *Streptomyces*. The piperidine derivatives 2-((1*E*,3*E*)-1,3-pentadienyl)piperidine (**20**) and 2-((1*E*,3*E*)-1,3-pentadienyl)piperidin-4-ol (SS20846A, **21**, Figure 5) have been reported from other streptomycetes [8,22–23]. They constitute hydrogenated analogs of the streptopyridines and occur together with streptazolin (**5**).

We then tested whether the streptopyridines can also be detected under conventional cultivation and isolation procedures. Therefore, strain FORM5 was grown as liquid culture. Headspace analysis revealed the production of the streptopyridines also under these conditions. The liquid cultures were either extracted with ethyl acetate (E-extract) or filtered over Oasis adsorption material. The latter was than extracted with ethyl acetate (O-extract). These extracts were analyzed by GC–MS. While the E-extract did not show the presence of any streptopyridine, they were present as trace components in the O-extract. Both extracts showed two major constituents, one with a molecular mass of 151 u (C_10_H_17_N, HRMS found 151.13656, calcd 151.13605), and the other with a molecular mass of 207 u (C_11_H_13_NO_3_, HRMS found 207.09056, calcd 207.08949). Although the compounds were not isolated, the mass spectral data and the previous reports of this class of compounds from *Streptomyces* led us to conclude that these compounds are indeed streptazolin (**5**, molecular mass 207) and 2-(penta-1,3-dien-1-yl)piperidine (**20**, molecular mass 151, mass spectra and high resolution data see Supporting Information File 1). Both compounds are accompanied by diastereomers exhibiting the same mass spectrum but different retention times. Minor components of the extracts could be tentatively assigned by their mass spectra to be the hydroxypiperidine **21**, occurring again as a pair of diastereomers, and streptazone D (**4**). The mass spectrum of **4** matches the published data [8]. Streptazone B_1_/B_2_ and/or C elute as broad peak from the GC column; a discrimination basing on the mass spectrum is not possible.

Several additional N-containing compounds occur in the extracts, some of them can be tentatively identified basing on their mass spectra. Two compounds exhibiting a small molecular ion at *m/z* 153 and a dominating base peak at *m*/*z* 84 are likely 2-(3-pentenyl)piperidines (**22**, mass spectrum see Supporting Information File 1). The ion *m*/*z* 84 is typical for 2-alkylpiperidines [24] and cannot be formed in piperidine **20** because of the adjacent double bond. The 3-pentenyl side chain is also supported by a small ion series at *m*/*z* 94 and 108 and present in streptopyridine E (**8**) and streptenols A (**28**) and B, biosynthetic precursors of this compound family (see below). Another compound, only present in the O-extract exhibits a molecular mass of 149 u, 2 units less than the major compound **20**. The mass spectrum (see Supporting Information File 1) resembles that of **20**. The additional double bond resides most likely at the nitrogen, arriving at the 1-piperideine structure **24**. A similar structure has the antibiotic nigrifactin (**26**), a hexaketide produced by *S. nigrifaciens*, differing only in the length of the side chain [25]. Piperideine **24** lacks a N–H and might get lost during work-up of the E-extract, contrary to the other alkylpiperidines. It can be detected as trace component in the headspace extract as well (see Table 1). As with piperidines **20** and **21**, a hydroxypiperideine **25** seems to be present (Figure 5, mass spectrum see Supporting Information File 1). Its oxidation product is streptazone D (**4**) in which the ring double bond moves into conjugation with the carbonyl group by imine–enamine tautomerisation. Finally compound **23** is produced, a derivative of piperidine **21** with an additional hydroxy group with unknown location in the ring. Compounds **22**–**25** (Figure 5) as well as the streptazolin isomer have not been reported from nature before.

**Figure 6:**
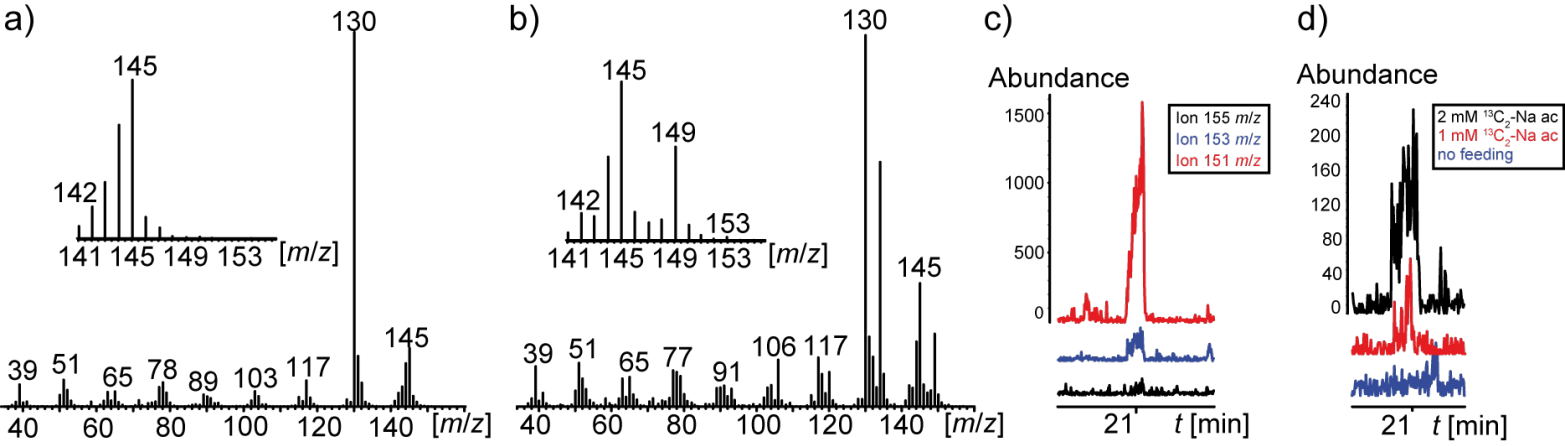
a) Mass spectrum of streptopyridine A (**12**), b) mass spectrum of **12** after feeding of 2 mM ^13^C_2_-sodium acetate, c) single ion chromatogram of 151, 153, 155 *m*/*z* after feeding with 2 mM ^13^C_2_-sodium acetate; d) single ion chromatogram of the 153 *m*/*z* molecular ion after feeding with 2 mM ^13^C_2_-acetate, 1 mM sodium ^13^C_2_-acetate, and a non fed culture as comparison. The ions at *m/z* 151, 153, and 155 indicate the incorporation of three, four, or five ^13^C_2_-acetate units.

We then tested whether the streptopyridines are biosynthetically produced via a polyketide sequence similar to that reported for streptazolin, involving a pentaketide precursor [13], or whether a specific pyridine precursor, e.g., pipecolic acid, with chain elongation was used. Feeding experiments with ^13^C_2_-sodium acetate showed incorporation of up to five acetate units by GC–MS, indicated by a mass shift of 10 amu for the molecular ion, from *m/z* 145 to *m/z* 155 (Figure 6a and 6b). Because of the dilution with unlabelled acetate the ^13^C_10_ isotopomer is of low abundance (Figure 6c). Nevertheless, the dose dependent incorporation of the acetate units clearly showed the presence of this isotopomer. Feeding of 2 mM sodium ^13^C_2_-acetate to agar plate cultures showed double incorporation compared to 1 mM sodium ^13^C_2_-acetate (Figure 6d). The results hint to a biosynthesis of the streptopyridines via the polyketide pathway.

**Scheme 2:**
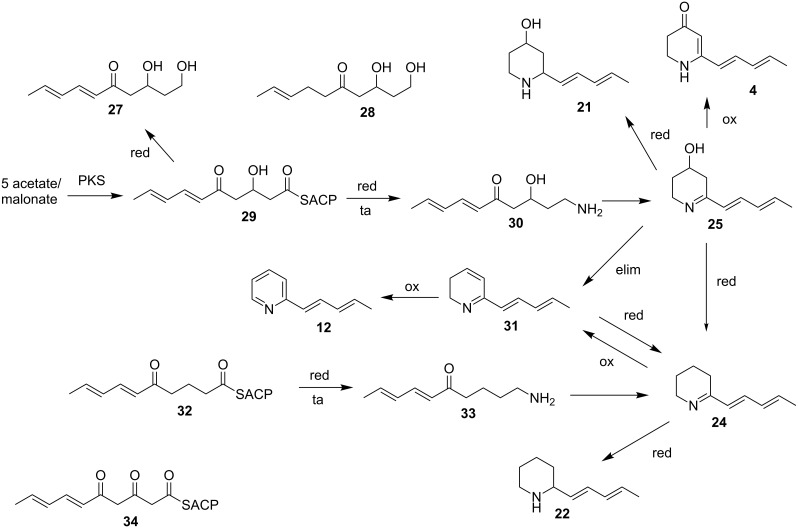
Proposed biosynthesis of the streptopyridines. PKS: polyketide synthase; red: reduction; ta: transamination; ox: oxidation; elim: elimination.

The proposed biosynthesis of the streptopyridines is shown in Scheme 2. The streptopyridines might be as well as the streptazones, streptenols (**27**) and piperidinols (**20**, **21**) [8–9] precursors or side products in the biosynthesis of streptazolin (**5**) [8]. The biosynthesis of **5** has been investigated earlier [13]. Mayer and Thiericke proposed a pentaketide precursor **34** that is transformed into the amine **33**. This amine is *N*-carboxylated, cyclized, and further processed to form **5** [13].

The streptopyridines might similarly be formed from the pentaketide **29**, showing the reduction status occurring in the previously isolated streptenols A and C (**27**) [9]. Reduction and transamination leads to the amine **30** that cyclizes to the piperideine **25**. Elimination of water leads to the dihydropyridine **31** that is oxidized to the major streptopyridine **12** possibly by spontaneous autoxidation in air. Isomerization of the double bonds during this pathway may lead to the isomers **9**–**11**. Reduction of **25** would lead to piperidine SS 20846A (**21**) [9,23]. Oxidation of intermediate **25** can also form streptazone D (**4**) by oxidation which would concomitantly shift the double bond into conjugation to the carbonyl group. Reductive removal of the hydroxy group, e.g., via **31** followed by double bond reduction may lead to piperideine **24**, a precursor of the major liquid phase component **22**. Alternatively, this compound might originate from the reduced PKS precursor **32** that is transformed via **33** into **24**. Piperidine **22** is then obtained via imine reduction as described. On the other hand, this pathway may also be the entry into the streptopyridine formation by oxidation of **24** to, e.g., **31**, followed by final oxidation.

A 3-pentenyl side chain as found in streptopyridine **8** and other derivatives occurs also in streptenol A (**28**), isolated from *S. luteogriseus* FH-S 1307 [9], *S. fimbriatus* [26], *S. cirratus* [27], and *Streptomyces* sp. HS-HY-045 [28]. This might indicate that the double bond hydrogenation occurs already during the pentaketide biosynthesis in these cases. Similarly, additional hydrogenation leads to the saturated alkylpyridine **7** and a tetraketide to propylpyridine (**6**).

**Figure 7:**
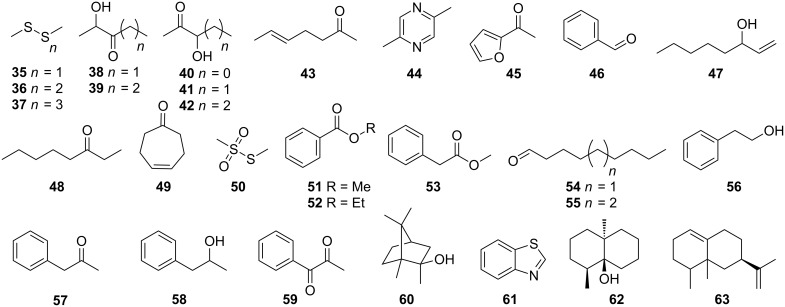
Compounds detected in the headspace of *Streptomyces* sp. FORM5.

In the bouquet of the headspace extract of *Streptomyces* strain FORM5 several other compounds besides the pyridines **6**–**12** were identified (Figure 7, Table 1). The most abundant of these were dimethyl disulfide (**35**), accompanied by other sulfur components as dimethyl trisulfide (**36**), dimethyl tetrasulfide (**37**), and *S*-methyl methanethiosulfonate (**50**). The hydroxyketones acetoin (**40**), and longer variants **38**, **39**, **41**, and **42** occur often in bacteria and are precursors of alkylated pyrazines [29] like 2,5-dimethylpyrazine (**44**). Aldehydes, ketones, and aromatic compounds commonly found as volatiles from bacteria were present in trace amounts: 5-hepten-2-one (**43**), 2-acetylfuran (**45**), 3-octanone (**48**), nonanal (**54**), decanal (**55**), 1-phenyl-2-propanone (**57**), 1-phenyl-1,2-propandione (**59**), benzaldehyde (**46**), methyl benzoate (**51**), 2-phenylethanol (**56**), 1-phenyl-2-propanol (**58**), ethyl benzoate (**52**), and methyl 2-phenylacetate (**53**) [2]. Of special interest is 5-hepten-2-one (**43**) that may be a shunt product of the streptopyridine E biosynthesis: It contains a 3-pentenyl chain motif adjacent to a carbonyl group, thus resembling the structural requirements of a possible streptopyridine E biosynthesis that follows the logic as presented in Scheme 2. Cyclohept-4-enone (**49**) has recently been reported from several actinomycetes [2]. Some terpenes could be identified as well. 2-Methylisoborneol (**60**) and geosmin (**62**) are commonly produced by *Streptomyces* and other actinomycetes [3,30]. In addition, the sesquiterpene valencene (**63**) was found, but the amount of terpenes released is quite low compared to other streptomycetes. Some nitrogen containing trace components could not be identified. In the E- and O-extracts similarly other nitrogen containing trace components occurred, although the small amounts excluded their identification.

**Table 1:** Volatile compounds identified in the headspace extract of *Streptomyces* strain FORM5. The amounts of the compounds are given as 0–2% (x), 2–8% (xx), >8% (xxx) relative to the largest peak area in the total ion chromatogram. The identification of the compound based on comparison of mass spectrum to a data base spectrum (ms), comparison of retention index to a published retention index on the same or similar GC fused silica capillary column (ri), and/or based on comparison to a synthetic or commercially available reference compound (std).

GC^a^	compound^b^	*I* (exp.)	*I* (lit.)^c^	Ident.	FORM5

	acetoin (**40**)	n.d.^d^		ms, std	xx
a	dimethyl disulfide (**35**)	n.d.		ms, std	xx
b	2-hydroxypentan-3-one (**38**)	829	822^*^	ms, ri	xx
	3-hydroxypentan-2-one (**41**)	833	838	ms, ri	x
c	5-hepten-2-one (**43**)	909		ms	x
d	2-hydroxyhexan-3-one (**39**)	910		ms	x
	3-hydroxyhexan-2-one (**42**)	914		ms	x
e	2,5-dimethylpyrazine (**44**)	925	925	ms, ri, std	x
f	2-acetylfuran (**45**)	925	923	ms, ri, std	x
g	benzaldehyde (**46**)	977	978	ms, ri, std	x
h	dimethyl trisulfide (**36**)	979	978	ms, ri, std	x
i	1-octen-3-ol (**47**)	987	987	ms, ri, std	x
j	3-octanone (**48**)	994	994	ms, ri, std	x
k	2-propylpyridine (**6**)	1000	1001	ms, ri, std	x
l	cyclohept-4-enone (**49**)	1023	1024	ms, ri, std	x
n	*m/z* = 118, 105, 77, 51, 39	1082			x
o	*S*-methyl methanethiosulphonate (**50**)	1083	1083	ms, ri	x
	methyl benzoate (**51**)	1108	1104	ms, ri, std	x
p	nonanal (**54**)	1116	1116	ms, ri, std	x
	*m/z* = 106, 135, 79	1128			x
q	2-phenylethanol (**56**)	1129	1129	ms, ri, std	x
	1-phenyl-2-propanone (**57**)	1143		ms	x
	1-phenyl-2-propanol (**58**)	1147		ms	x
	1-phenyl-1,2-propandione (**59**)	1183	1186	ms, ri,	x
	methyl 2-phenylacetate (**53**)	1189	1187	ms	x
	2-methylisoborneol (**60**)	1199	1201	ms, ri, std	x
r	2-pentylpyridine (**7**)	1205	1205	ms, ri, std	x
s	(*E*)-2-(pent-3-enyl)pyridine (**8**)	1207	1208	ms, ri, std	xxx
t	*m/z* = 132, 93, 118, 41, 106	1212			x
u	decanal (**55**)	1216	1216	ms, ri, std	x
v	*m/z* = 118, 93, 46, 130, 52	1219			x
	dimethyl tetrasulfide (**37**)	1233	1234	ms, ri, std	x
	benzothiazole (**61**)	1246	1246	ms, ri, std	x
	ethyl 2-phenylacetate	1256	1252	ms	x
w	*m/z* = 118, 147, 132, 91, 51	1283			x
x	*m/z* = 79, 104, 133, 51, 117	1321			x
y	2-((1*Z*,3*E*)-penta-1,3-dienyl)pyridine (**9**)	1327	1334	ms, ri, std	xx
z	2-((1*Z*,3*Z*)-penta-1,3-dienyl)pyridine (**10**)	1352	1358	ms, ri, std	xxx
aa	2-((1*E*,3*Z*)-penta-1,3-dienyl)pyridine (**11**)	1362	1363	ms, ri, std	xx
ab	2-((1*E*,3*E*)-penta-1,3-dienyl)pyridine (**12**)	1366	1368	ms, ri, std	xxx
ac	geosmin (**62**)	1429	1430	ms, ri, std	x
ad	*m/z* = 105, 120, 91, 204, 176	1465			x
ae	valencene (**63**)	1499	1498	ms, ri, std	x
af	*m/z* = 148, 163, 120	1529		ms	x

^a^Compound assignment refers to Figure S2 (Supporting Information File 1), ^b^artefacts found in both control and inoculated samples are not listed, ^c^taken from NIST Chemistry WebBook [14] or our own data base, ^*^DB-5, A: artifact; ^d^not determined.

The streptopyridines showed only weak antibacterial and cytostatic activity (R. Müller, pers. commun.), similar to that described earlier by us for 2-pentylpyridine [31]. The analysis of the 16S-RNA revealed strain FORM5 to be a *Streptomyces*. It showed 99% sequence similarity to *Streptomyces griseosporus* (R. Müller, pers. commun.).

The two analytical methods used in the current work are obviously orthogonal to each other. They can be used on the same culture. After initial sampling of the headspace of a liquid culture, the culture can be separated from the cells and extracted as described. This consecutive approach allows a broad overview on the metabolites. The E- or O-extracts can be analyzed by HPLC–MS and GC–MS. Small, basic compounds as the piperidines are released from the liquid medium and can be detected only by the extraction methods. Their basicity and/or their ability to interact by hydrogen bonding with water obviously prevent release from the water phase in substantial amounts. Volatile compounds like the streptopyridines are less soluble, have a lower basicity and can be detected as major compounds in the headspace. Both analytical methods have a different analytical window and complement each other.

The detected volatiles fall into different groups. Most of the compounds listed in Table 1 can be regarded as volatiles commonly produced by various bacteria. That does not imply that most bacteria release them, but that these volatiles often occur when bacteria are analyzed. These compounds form a chemical structure space of volatiles released by bacteria and their number is limited, although this space is certainly only partially explored. Another group of volatile compounds forming a structure space is already known from the green part of plants (excluding flowery parts). The plant structure space is different from that emerging for bacteria. The bacterial structure space includes acetoins and aromatic aldehydes, esters and ketones, aliphatic compounds, sulfur compounds, but also 4-cycloheptenone that we found in several other bacteria (unpublished results). The terpenes geosmin and methylisoborneol are typical terpenes of the actinomycetes. Various sesquiterpene cyclases occur in the streptomycetes [3,30,32] and give rise to a wide variety of sesquiterpenes, in this case valencene.

The only strain specific compounds are the streptopyridines. They have not been reported from other strains and their biosynthesis seems tightly connected to the biosynthesis of streptazolin. A similar case has been reported for specific volatile butenolides released by streptomycetes producing the antibiotic antimycin [33] and for other strains investigated by us. The analysis of volatiles and detection of unique compounds may thus be used as a method to select microbial strains for the detection of non-volatile biologically active compounds. Alternatively, two independent gene clusters may be responsible for streptazolin and streptopyridines.

The production of strain specific compounds connected to, e.g., antibiotic biosynthesis might also have important ecological consequences. As an example, streptazolin producing streptomycetes have been isolated from mud-dauber wasps [34]. They have been postulated as antibiotic-producing symbionts of the wasps. If this is true, they should also produce specific volatiles as the streptopyridines and add to the odor of the insect. Given the many different bacteria living on the insect, a specific odor bouquet of the insect would arise, assembled by insect derived compounds and bacterial volatiles, finally carrying information on the individual symbiont composition. The symbiont composition might affect the fitness and, if it can be perceived by interacting partners, may in the end influence the behavior of the insects, e.g., mate choice or aggression.

## Conclusion

In conclusion, the investigation of the headspace extract of *Streptomyces* strain FORM5 revealed the occurrence of new 2-alkylpyridines that are structurally related to streptazolin and 2-pentadienylpiperidines produced by this strain. While they are the major compounds in the volatile bouquet, they occur only in minor amounts in the liquid phase. In contrast, the major compounds streptazolin and 2-pentadienylpiperidine do not occur in the headspace, thus proving the necessity to use orthogonal analytical methods to assess the full metabolic potential of a microorganism.

## Experimental

### General experimental procedures

Reagents and solvents were purchased from Sigma-Aldrich Chemie GmbH (Steinheim, Germany) and Acros Organics (Geel, Belgium) and used without further purification. Solvents were distilled before use and, if necessary, dried using standard procedures. All non aqueous reactions were performed under an inert atmosphere (N_2_) in flame-dried flasks. Purification of the synthetic products was carried out by flash chromatography using Merck silica gel 60 (70–200 mesh). Thin-layer chromatography was performed with 0.2 mm pre-coated polyester sheets (Polygram SIL (G/UV254), Macherey-Nagel). NMR spectra were obtained on either a Bruker DRX-400 (400 MHz) or an AV III-400 (400 MHz) spectrometer and were referenced against TMS (δ = 0.00 ppm) for ^1^H NMR and CHCl_3_ (δ = 77.16 ppm) for ^13^C NMR. GC–MS analysis were performed on an Agilent 7890A gas chromatograph connected to an Agilent 6975 C inert mass detector fitted with a BPX-5 fused silica capillary column (25 m, 0.25 mm i.d., 0.25 μm film). Conditions were as follows: inlet pressure 67 kPa, He 23.3 mL/min, injection volume 1 µL, transfer line 300 °C, injector 250 °C, electron energy 70 eV. The gas chromatograph was programmed as follows: 5 min at 50 °C, then increasing with 5 °C/min to 320 °C. Linear retention indices were determined from a homologous series of *n*-alkanes (C8–C32). Compounds were identified by comparison of mass spectra to database spectra (Wiley 7, NIST 08 and our own created from synthesized reference compounds), by comparison of the retention index data to standards (own database and NIST Chemistry WebBook (2013) [14]) and by synthesis of reference compounds.

#### Organism and analysis

*Streptomyces* strain FORM5 was isolated from a soil sample collected in Formentera (Spain) and is deposited at the Institute of Organic Chemistry in Göttingen [8]. It was cultivated in 10 mL SM-media (20 g/L mannitol, 20g/L soy flour, 4 mL/L 2.5 M magnesium chloride solution, 20 g/L agar only for plates) at 28 °C for 3 days. SM-agar plates were then inoculated with 300 µL of the preculture and cultivated for 6 days at 28 °C. Then the culture was analyzed by closed-loop stripping analysis at room temperature [7]. In this system, air is continuously pumped (MB-21E, Senior Flextronics, USA) through the closed system that contains an activated charcoal filter (Chromtech GmbH, Idstein, Precision Charcoal Filter, 5 mg) and the agar plate or liquid culture for 24 hours. The filter was then extracted by rinsing 3× with 15 µL dichloromethane (≥99.8%, Merck, Germany) and the resulting headspace extract was analyzed by GC–MS. The experiment was repeated at least three times. The SM-medium was analyzed without inoculation as control. Liquid cultures were analyzed similarly by the CLSA method. After the collection of volatiles the liquid phase was analyzed by two different methods. E-extract: The culture media was centrifuged for 20 min at 4 °C and the supernatant was extracted 3 times with 50 mL CH_2_Cl_2_. The extracts were dried with MgSO_4_, concentrated under reduced pressure and analyzed by GC–MS. O-extract A Oasis^®^ HLB cartridge (Waters) was prewashed with 2 column volumes ethyl acetate and conditioned with 2 column volumes water. The culture media was centrifuged for 20 min at 4 °C and the supernatant (100 mL) filtered through the cartridge. The cartridge was extracted with 3 column volumes ethyl acetate, the eluent was dried with MgSO_4_, concentrated under reduced pressure, and finally subjected to GC–MS analysis. For feeding experiments on the biosynthesis of compound **7** the SM-media was enriched with sodium ^13^C_2_-acetate (ISOTEC) in 1 mM (1.7 mg, 0.02 mmol, in 20 mL medium) and 2 mM (3.4 mg, 0.04 mmol, in 20 mL medium) followed by collection of the volatiles by CLSA.

#### Synthesis of reference compounds

The compounds **6**–**8** were synthesized after the standard procedure for iron-catalyzed aryl–alkyl cross-coupling described by Fürstner et al [17].

2-Propylpyridine (**6**): Yield (1.01 g, 8.35 mmol, 61%); *R*_f_ (pentane/Et_2_O 5:1) 0.2; UV (CH_2_Cl_2_) λ_max_ (log ε): 257 (5.77), 262 (5.78) nm; IR (diamond) ν_max_: 2960, 2832, 2871, 1590, 1569, 1472, 1434, 1149, 1051, 993, 751 cm^−1^; ^1^H NMR (CDCl_3_, 400 MHz) δ ppm 0.97 (t, *J* = 7.4 Hz, 3H, CH_3_), 1.76 (sxt, *J* = 7.5 Hz, 2H, CH_2_), 2.74–2.79 (m, 2H, CH_2_), 7.07–7.11 (m, 1H, CH), 7.14 (dd, *J* = 7.8, 0.5 Hz, 1H, CH), 7.58 (tdd, *J* = 7.6, 1.9, 0.9 Hz, 1H, CH), 8.52 (dt, *J* = 4.89, 0.94 Hz, 1H, CH); ^13^C NMR (CDCl_3_, 100 MHz) δ ppm 13.8 (CH_3_), 23.1 (CH_2_), 40.4 (CH_2_), 120.8 (CH), 122.7 (CH), 136.1 (CH), 149.2 (CH), 162.3 (C); EIMS *m*/*z*: 121 [M]^+^ (2), 120 (9), 106 (29), 93 (100), 78 (12), 65 (18), 51 (15), 39 (18); HREIMS *m*/*z*: calcd for C_8_H_11_N, 121.0891; found, 121.0888; GC (BPX-5) *I* = 1001.

2-Pentylpyridine (**7**): Yield (827 mg, 5.6 mmol, 56%); *R*_f_ (pentane/Et_2_O 5:1) 0.2; UV (CH_2_Cl_2_) λ_max_ (log ε): 257 (6.28), 262 (6.29) nm; IR (diamond) ν_max_: 2955, 2928, 2858, 1590, 1472, 1434, 1148, 993 cm^−1^; ^1^H NMR (CDCl_3_, 400 MHz) δ ppm 0.86–0.93 (m, 3H, CH_3_), 1.29–1.40 (m, 4H, 2× CH_2_), 1.68–1.78 (m, 2H, CH_2_), 2.74–2.82 (m, 2H, CH_2_), 7.08 (ddd, *J* = 7.5, 4.9, 1.1 Hz, 1H, CH), 7.13 (dd, *J* = 7.8, 1.0 Hz, 1H, CH), 7.53–7.60 (m, 1H, CH), 8.50–8.55 (m, 1H, CH); ^13^C NMR (CDCl_3_, 100 MHz) δ ppm 13.9 (CH_3_), 22.5 (CH_2_), 29.5 (CH_2_), 31.5 (CH_2_), 38.4 (CH_2_), 120.7 (CH), 122.6 (CH), 136.1 (CH), 149.1 (CH), 162.5 (C); EIMS *m*/*z*: 149 [M]^+^ (2), 93 (100), 120 (25), 106 (30), 92 (14), 78 (14), 65 (16), 51 (10), 39 (14); HREIMS *m*/*z*: calcd for C_10_H_15_N, 149.1204; found, 149.1203; GC (BPX-5) *I* = 1205.

2-((*E*)-Pent-3-en-1-yl)pyridine (**8**): Yield (326 mg, 2.2 mmol, 16%); *R*_f_ (pentane/Et_2_O 5:1) 0.24; UV (CH_2_Cl_2_) λ_max_ (log ε): 257 (5.73), 262 (5.74) nm; IR (diamond) ν_max_: 3009, 2918, 2854, 1590, 1569, 1474, 1434, 1148, 1051, 966, 750 cm^−1^; ^1^H NMR (CDCl_3_, 400 MHz) δ ppm 1.62–1.64 (m, 3H, CH_3_), 2.38–2.44 (m, 2H, CH_2_), 2.82–2.85 (m, 2H, CH_2_), 5.41–5.52 (m, 2H, 2× CH), 7.07–7.10 (m, 1H, CH), 7.13 (d, *J* = 7.8 Hz, 1H, CH), 7.57 (td, *J* = 7.6, 1.9 Hz, 1H, CH), 8.51–8.53 (m, 1H, CH); ^13^C NMR (CDCl_3_, 100 MHz) δ ppm 17.5 (CH_3_), 32.4 (CH_2_), 38.1 (CH_2_), 120.6 (CH_2_), 122.4 (CH_2_), 125.2 (CH), 129.9 (CH), 135.8 (CH), 148.8 (CH), 161.4 (C); EIMS *m*/*z*: 147 [M]^+^ (17), 146 (29), 133 (11), 132 (100), 119 (17), 118 (30), 117 (47), 106 (25), 93 (77), 79 (17), 78 /14), 65 (18), 51 (13), 39 (19); HREIMS *m*/*z*: calcd for C_10_H_13_N, 147.1048; found, 147.1054; GC (BPX-5) *I* = 1208.

The synthesis of compounds **9** and **12** was achieved by Wittig reaction starting with the synthesis of crotyltriphenylphosphonium bromide (**18**). Crotyl bromide (**17**, 2.23 g, 16.52 mmol) was added to a mixture of triphenylphosphine (3.67 g, 14 mmol) in THF and stirred under reflux overnight. THF was removed under reduced pressure and the resulting solid was dissolved in a mixture of CH_2_Cl_2_/MeOH (10:1). Column chromatography on silica gel with CH_2_Cl_2_/MeOH (15:1, *R*_f_ = 0.25) yielded **18** (5.12 g, 12.9 mmol, 92%) as a white solid. ^1^H NMR (CDCl_3_, 200 MHz) δ ppm 1.35–1.42 (m, 3H, CH_3_), 1.58–1.65 (m, 3H, CH_3_), 4.54–4.69 (m, 4H, 2× CH_2_), 5.24–5.39 (m, 2H, 2× CH), 5.87–6.04 (m, 2H, 2× CH), 7.65–7.92 (m, 30H, 30× CH); ^13^C NMR (CDCl_3_, 50 MHz) δ ppm 18.1 (CH_3_), 18.2 (CH_3_), 27.2 (CH_2_), 28.2 (CH_2_), 114.6 (CH), 114.8 (CH), 117.0 (3× C), 118.7 (3× C), 130.0 (6× CH), 130.3 (6× CH), 133.6 (6× CH), 133.8 (6× CH), 134.8 (3× CH), 134.9 (3× CH), 137.4 (CH), 137.7 (CH); ^31^P NMR (CDCl_3_, 80 MHz) δ ppm 21.4 (s), 21.6 (s). At room temperature an ethereal phenyllithium solution (1.63 mL, 15.5 mmol) was added to a mixture of **18** (6.15 g, 15.5 mmol) in 30 mL dry THF and 45 mL dry diethyl ether. After stirring for 20 min at rt the mixture was cooled down to −78 °C and pyridine-2-carbaldehyde (**19**, 1.66 g, 15.5 mmol) was added. Afterwards the mixture was allowed to warm to −30 °C in 2 h and again phenyllithium solution (1.63 mL, 15.5 mmol) was added. Then the mixture was cooled down again to −78 °C and potassium *tert*-butoxide (2.61 g, 23.25 mmol) was added. Overnight the mixture was allowed to warm to rt and quenched with dest. water. The aqueous layer was extracted two times with diethyl ether. The combined organic layers were dried with MgSO_4_ and the solvent was removed under reduced pressure. Column chromatography on silica gel with pentane/diethyl ether (5:1) yielded **9** (0.36 mmol, 53 mg, *R*_f_ = 0.3, 3%) and **12** (2.22 mmol, 322 mg, *R*_f_ = 0.25, 14%) as yellow oils (crude yield was 62%).

2-((1*Z*,3*E*)-Penta-1,3-dien-1-yl)pyridine (**9**): UV (CH_2_Cl_2_) λ_max_ (log ε): 271 (6.66), 300 (6.71), 308 (6.72) nm; IR (diamond) ν_max_: 3006, 2962, 2911, 1641, 1582, 1558, 1469, 1431, 1149, 990, 833, 797, 741 cm^−1^; ^1^H NMR (CDCl_3_, 400 MHz) δ ppm 1.85 (dd, *J* = 6.8, 1.7 Hz, 3H, CH_3_), 5.96 (dq, *J* = 15.2, 6.8 Hz, 1H, CH), 6.23 (d, *J* = 11.8 Hz, 1H, CH), 6.34 (dd, *J* = 11.5, 11.2 Hz, 1H, CH), 7.07 (dd, *J* = 7.5, 5.0 Hz, 1H, CH), 7.21 (d, *J* = 7.9 Hz, 1H, CH), 7.40 (dd, *J* = 15.2, 11.0 Hz, 1H, CH), 7.61 (td, *J* = 7.7, 1.9 Hz, 1H, CH), 8.60–8.62 (m, 1H, CH); ^13^C NMR (CDCl_3_, 100 MHz) δ ppm 18.5 (CH_3_), 121.0 (CH), 124.1 (CH), 125.5 (CH), 128.8 (CH), 134.1 (CH), 135.2 (CH), 136.0 (CH), 149.3 (CH), 156.9 (C); EIMS *m*/*z*: 145 [M]^+^ (9), 144 (12), 143 (6), 142 (5), 131 (10), 130 (100), 117 (5), 103 (4), 89 (3), 78 (5), 77 (4), 65 (3), 51 (4), 39 (3); HREIMS *m*/*z*: calcd for C_10_H_13_N, 145.0892; found, 145.0912; GC (BPX-5) *I* = 1334.

2-((1*E*,3*E*)-penta-1,3-dien-1-yl)pyridine (**12**): UV (CH_2_Cl_2_) λ_max_ (log ε): 272 (6.55), 300 (6.60), 308 (6.61) nm; IR (diamond) ν_max_: 3008, 2962, 2911, 1644, 1582, 1561, 1467, 1429, 1145, 989, 796, 755, 740 cm^−1^; ^1^H NMR (CDCl_3_, 400 MHz) δ ppm 1.82 (dd, *J* = 6.8, 2 Hz, 3H, CH_3_), 5.96 (dq, *J* = 15.7, 6.8 Hz, 1H, CH), 6.26 (dd, *J* = 15.1, 10.8 Hz, 1H, CH), 6.48 (d, *J* = 15.8 Hz, 1H, CH), 7.04 (dd, *J* = 7.5, 5.0 Hz, 1H, CH), 7.21 (dd, *J* = 15.1, 11.3 Hz, 1H, CH), 7.21 (d, *J* = 7.8 Hz, 1H, CH), 7.66 (td, *J* = 7.7, 1.9 Hz, 1H, CH), 8.51–8.53 (m, 1H, CH); ^13^C NMR (CDCl_3_, 100 MHz) δ ppm 18.3 (CH_3_), 121.3 (CH), 121.4 (CH), 128.9 (CH), 131.3 (CH), 133.0 (CH), 133.2 (CH), 136.1 (CH), 149.3 (CH), 155.8 (C); EIMS *m*/*z*: 145 (18) [M]^+^, 144 (14), 143 (6), 142 (5), 131 (10), 130 (100), 117 (6), 103 (4), 78 (5), 77 (4), 65 (3), 51 (5), 39 (3); HREIMS *m*/*z*: calcd for C_10_H_13_N, 145.0892; found, 145.0912; GC (BPX-5) *I* = 1368.

## Supporting Information

File 1 Total ion chromatograms of strain *Streptomyces* sp*.* FORM5, mass spectra, 16S-RNA data, and ^1^H and ^13^C NMR spectra of the synthetic compounds.
