# Supplementary material for: Streptopyridines, volatile pyridine alkaloids produced by Streptomyces sp. FORM5
Source: Beilstein J Org Chem. 2014 Jun 24;10:1421–32. doi: 10.3762/bjoc.10.146 (PMC4077424; doi:10.3762/bjoc.10.146)
Supplement: File 1 — Total ion chromatograms of strain Streptomyces sp. FORM5, mass spectra, 16S-RNA data, and 1H and 13C NMR spectra of the synthetic compounds. [file Beilstein_J_Org_Chem-10-1421-s001.pdf]

Supporting Information

for

Streptopyridines, volatile pyridine alkaloids produced by *Streptomyces* sp. FORM5

Ulrike Groenhagen[1], Michael Maczka[1], Jeroen S. Dickschat[1] and Stefan Schulz[1,]*

Address: [1]Institut für Organische Chemie, Technische Universität Braunschweig, Hagenring 30, 38106 Braunschweig, Germany

Email: Stefan Schulz* - stefan.schulz@tu-braunschweig.de

*Corresponding author

Total ion chromatograms of strain *Streptomyces* sp. FORM5, mass spectra, 16S-RNA data, and ^{1}H and ^{13}C NMR spectra of the synthetic compounds

Streptomyces sp. FORM5, 16S-Sequenz

GACTTCGTCCCAATCGCCAGTCCCACCTTCGACAGCTCCCTCCCGCGAACGGGTTGGGCCACCGGCTT
CGGGTGTTACCGACTTTCGTGACGTGACGGGCGGTGTGTACAAGGCCCGGGAACGTATTCACCGCAGC
AATGCTGATCTGCGATTACTAGCGACTCCGACTTCATGGGGTCGAGTTGCAGACCCCAATCCGAACTG
AGACCGGCTTTTTGAGATTCGCTCCACCTCACGGTATCGCAGCTCATTGTACCGGCCATTGTAGCACG
TGTGCAGCCCAAGACATAAGGGGCATGATGACTTGACGTCGTCCCCACCTTCCTCCGAGTTGACCCCG
GCGGTCTCCCGTGAGTCCCCAGCACCACAAGGGCCTGCTGGCAACACGGGACAAGGGTTGCGCTCGTT
GCGGGACTTAACCCAACATCTCACGACACGAGCTGACGACAGCCATGCACCACCTGTACACCGACCAC
AAGGGGGCGCCCATCTCTGGACGTTTCCGGTGTATGTCAAGCCTTGGTAAGGTTCTTCGCGTTGCGTC
GAATTAAGCCACATGCTCCGCCGCTTGTGCGGGCCCCCGTCAATTCCTTTGAGTTTTAGCCTTGCGGC
CGTACTCCCCAGGCGGGGCACTTAATGCGTTAGCTGCGGCACGGACAACGTGGAATGTTGCCCACACC
TAGTGCCCACCGTTTACGGCGTGGACTACCAGGGTATCTAATCCTGTTCGCTCCCCACGCTTTCGCTC
CTCAGCGTCAGTATCGGCCCAGAGATCCGCCTTCGCCACCGGTGTTCCTCCTGATATCTGCGCATTTC
ACCGCTACACCAGGAATTCCGATCTCCCCTACCGAACTCTAGCCTGCCCGTATCGACTGCAGACCCGG
GGTTAAGCCCCGGGCTTTCACAACCGACGTGACAAGCCGCCTACGAGCTCTTTACGCCCAATAATTCC
GGACAACGCTTGCGCCCTACGTATTACCGCGGCTGCTGGCACGTAGTTAGCCGGCGCTTCTTCTGCAG
GTACCGTCACTTTCGCTTCTTCCCTGCTGAAAGAGGTTTACAACCCGAAGGCCGTCATCCCTCACGCG
GCGTCGCTGCATCAGGCTTTCGCCCATTGTGCAATATTCCCCACTGCTGCCTCCCGTAGGAGTCTGGG
CCGTGTCTCAGTCCCAGTGTGGCCGGTCGCCCTCTCAGGCCGGCTACCCGTCGTCGCCTTGGTGAGCC
GTTACCTCACCAACAAGCTGATAGGCCGCGGGCTCATCCTGCACCGCCGGAGCTTTACAGAACCAAGG
ATGCCCAAGGCTCTCATATCCGGTATTAGACCCCGTTTCCAGGGCTTGTCCCAGAGTGCAGGGCAGAT
TGCCCACGTGTTACTCACCCGTTCGCCACTAATCCCCACCGAAGTGGTTCATCGTTCGACTGCATGTG
TTAAGCACGCCGCCAGCGTTCGTCT

Sequence alignment results

Sequences producing significant alignments:

Accession	Description	Max score	Total score	Query coverage	E value	Max ident
AB184419.1	Streptomyces griseosporeus gene for 16S rRNA, partial sequence, strai	2606	2606	99%	0.0	99%
AB184361.2	Streptomyces viridis gene for 16S rRNA, partial sequence, strain: NBRC	2599	2599	99%	0.0	98%
GQ214030.1	Streptomyces sp. ZG0650 16S ribosomal RNA gene, partial sequence	2593	2593	99%	0.0	98%
GU130108.1	Streptomyces sp. 172618 16S ribosomal RNA gene, partial sequence	2582	2582	99%	0.0	98%
GQ214028.1	Streptomyces sp. ZG0737 16S ribosomal RNA gene, partial sequence	2582	2582	99%	0.0	98%
AB184648.1	Streptomyces cinereospinus gene for 16S rRNA, partial sequence, strair	2580	2580	99%	0.0	98%
AB184849.1	Streptomyces coeruleorubidus gene for 16S rRNA, partial sequence, str	2577	2577	99%	0.0	98%
GQ924534.1	Streptomyces sp. ACT-0094 16S ribosomal RNA gene, partial sequence	2571	2571	99%	0.0	98%
FJ429553.1	Uncultured Streptomyces sp. clone 2440 16S ribosomal RNA gene, parti	2571	2571	99%	0.0	98%
EF063473.1	Streptomyces sp. 558(1) 16S ribosomal RNA gene, partial sequence	2571	2571	99%	0.0	98%
AB184584.1	Streptomyces thermocoerulescens gene for 16S rRNA, partial sequence	2571	2571	99%	0.0	98%
AB184877.1	Streptomyces iakyrus gene for 16S rRNA, partial sequence, strain: NBR	2569	2569	99%	0.0	98%
AB184504.1	Streptomyces kagoshimanus gene for 16S rRNA, partial sequence, strai	2569	2569	99%	0.0	98%
AY999873.1	Streptomyces griseosporeus strain AS 4.1840 16S ribosomal RNA gene,	2567	2567	98%	0.0	99%
FJ429558.1	Uncultured Streptomyces sp. clone 2509 16S ribosomal RNA gene, parti	2566	2566	99%	0.0	98%
EU741215.1	Streptomyces iakyrus strain 13667L 16S ribosomal RNA gene, partial se	2564	2564	99%	0.0	98%
EF056498.1	Streptomyces sp. 1A01629 16S ribosomal RNA gene, complete sequenc	2564	2564	99%	0.0	98%
AB184326.1	Streptomyces parvulus gene for 16S rRNA, partial sequence, strain: NB	2564	2564	99%	0.0	98%
EF063493.1	Streptomyces sp. P3562 16S ribosomal RNA gene, partial sequence	2562	2562	99%	0.0	98%
AJ781382.1	Streptomyces purpurascens 16S rRNA gene, type strain LMG 20526	2562	2562	99%	0.0	98%
HM367877.1	Streptomyces sp. Ank289 16S ribosomal RNA gene, partial sequence	2560	2560	99%	0.0	98%
GU433228.1	Streptomyces sp. ABRIINW 111 16S ribosomal RNA gene, complete seq	2560	2560	99%	0.0	98%
GQ925802.1	Streptomyces albogriseolus strain ABRIINW EA1145 16S ribosomal RNA	2560	2560	99%	0.0	98%
DQ663193.1	Streptomyces sp. 3176 16S ribosomal RNA gene, partial sequence	2560	2560	99%	0.0	98%
AB249954.1	Streptomyces malachitospinus gene for 16S rRNA, partial sequence, str	2560	2560	99%	0.0	98%
AB045888.1	Streptomyces purpurascens gene for 16S rRNA	2560	2560	99%	0.0	98%
AB045865.1	Streptomyces heteromorphus gene for 16S rRNA	2560	2560	99%	0.0	98%
FJ626660.1	Streptomyces sp. MH105 16S ribosomal RNA gene, partial sequence	2558	2558	99%	0.0	98%
EF012113.1	Streptomyces sp. 1A01536 16S ribosomal RNA gene, partial sequence	2558	2558	99%	0.0	98%
AB184859.1	Streptomyces purpurascens gene for 16S rRNA, partial sequence, strair	2558	2558	99%	0.0	98%

These data were provided by R. Müller (pers. commun.). The data were obtained with the method described in: Raju, R., Gromyko, O., Fedorenko, V., Luzhetskyy, A. and Müller, R., Oleaceran: A novel spiro[isobenzofuran-1,2'-naptho[1,8-bcfuran] isolated from a terrestrial *Streptomyces* sp, *Org. Lett.* **2013,** *15,* 3487–3489.

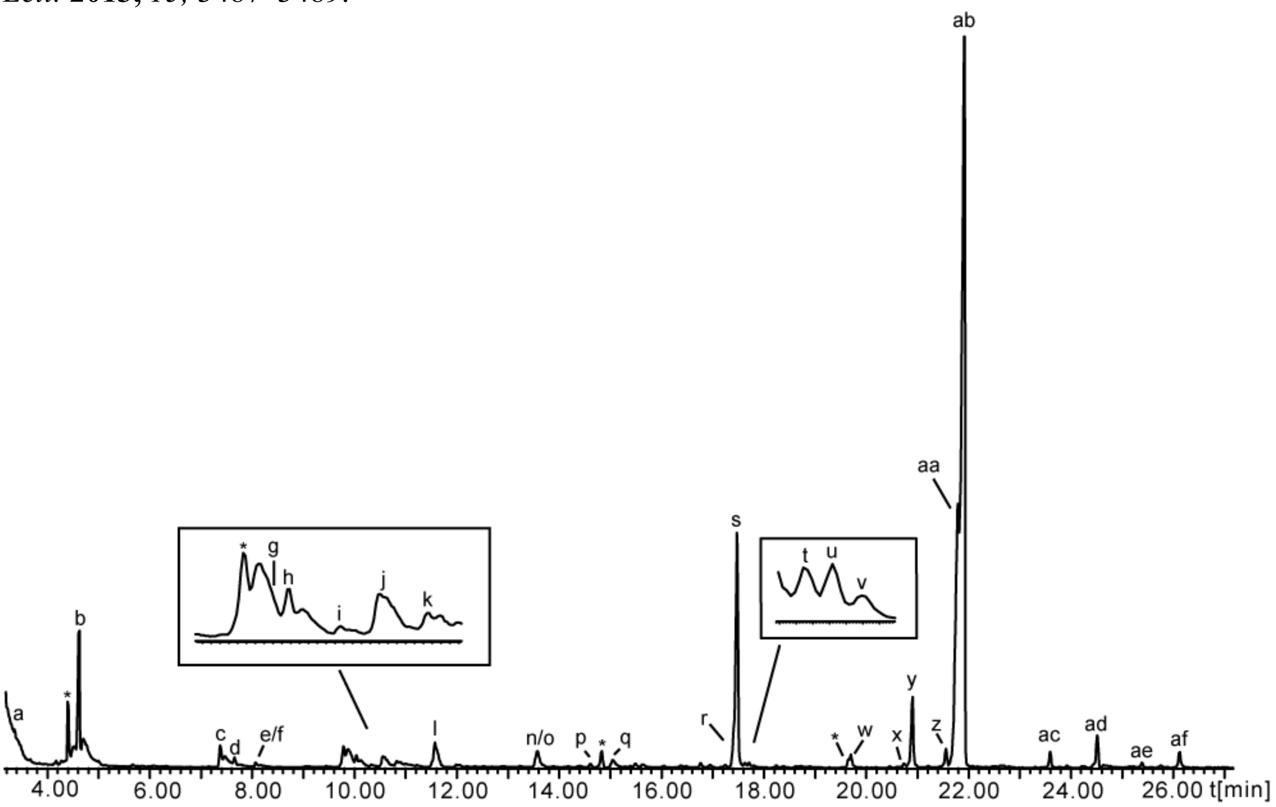

Figure S1: Total ion chromatogram of the headspace extract of *Streptomyces* sp. FORM5. Nomination refers to Table 1. Artifacts are marked with *.

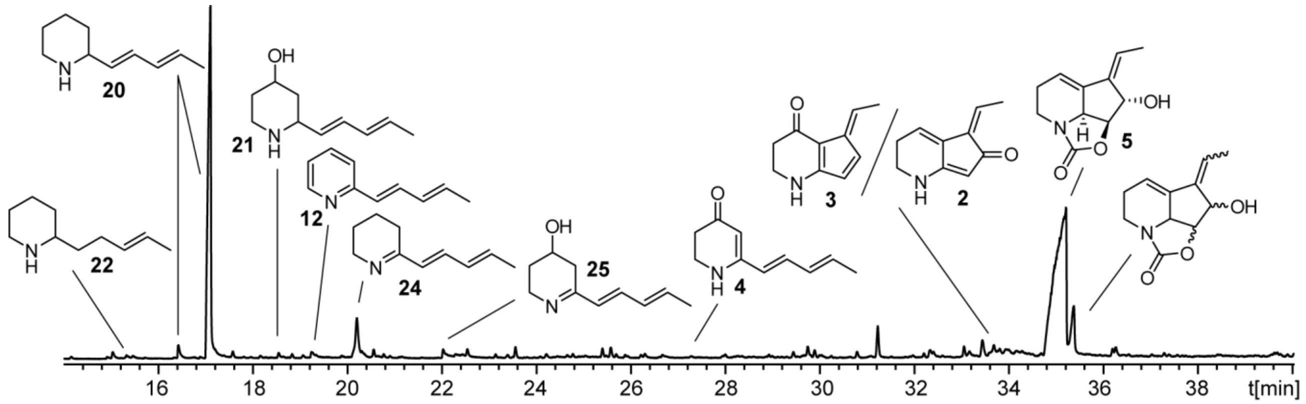

Figure S2: Total ion chromatogram of the O-extract of *Streptomyces* sp. FORM5.

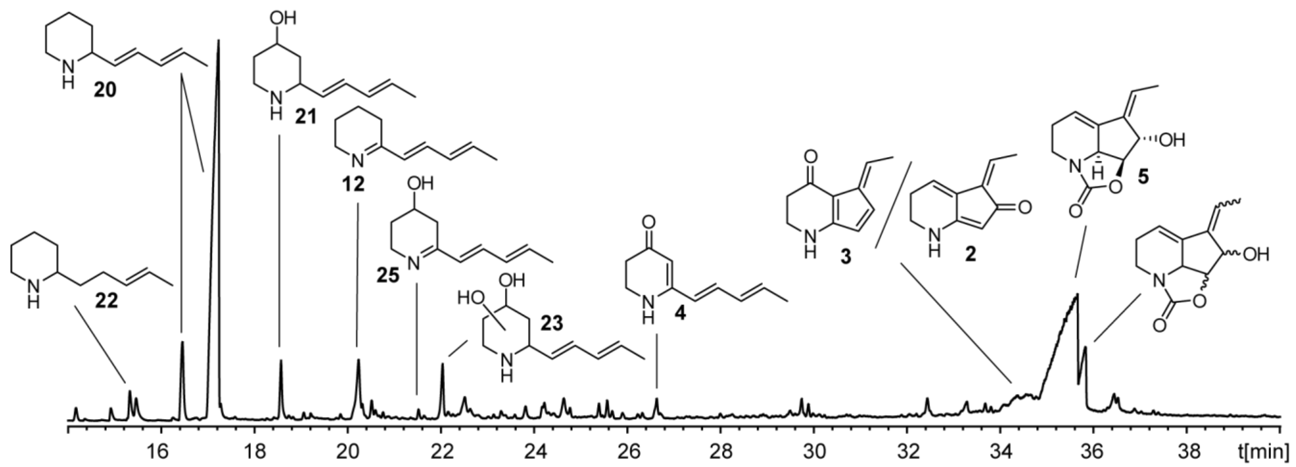

Figure S3: Total ion chromatogram of the E-extract of *Streptomyces* sp. FORM5.

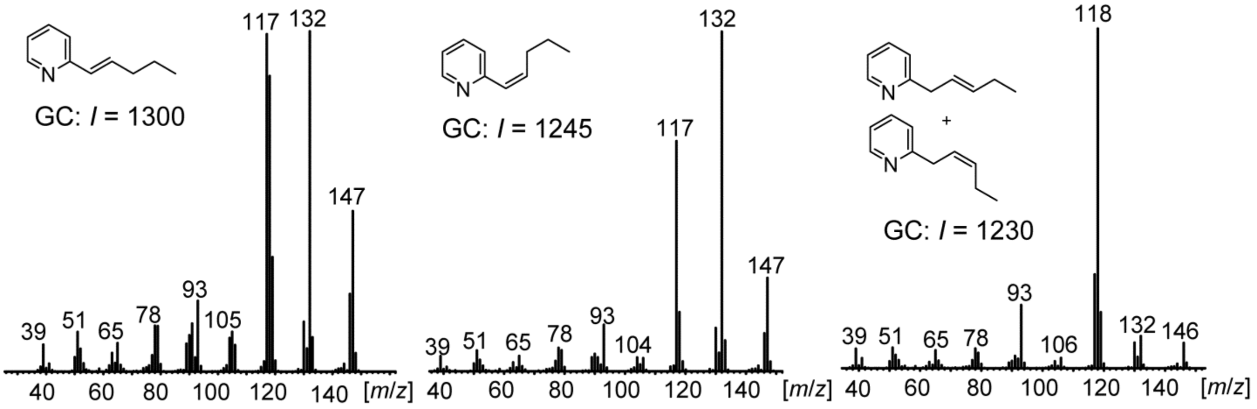

Figure S4: EI-Mass spectra of synthetic 2-pentenylpiperidines. *I*: retention index.

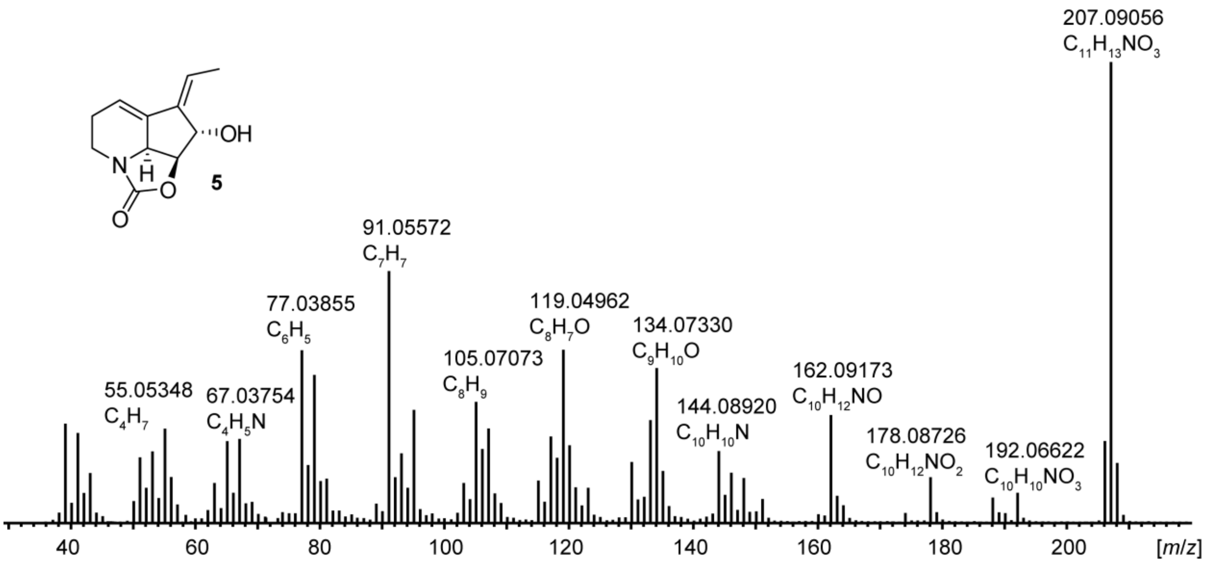

Figure S5: EI-Mass spectrum of streptazolin and high resolution MS data.

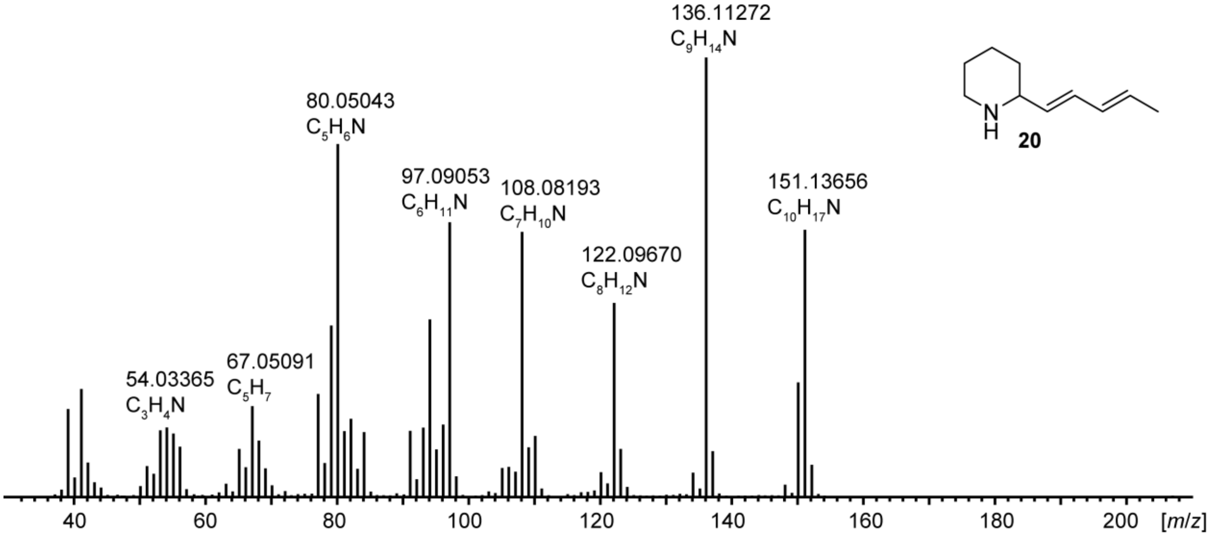

Figure S6: EI-Mass spectrum and high resolution MS data of 2-(1*E*,3*E*)-1,3-petadienyl)piperidine (**20**).

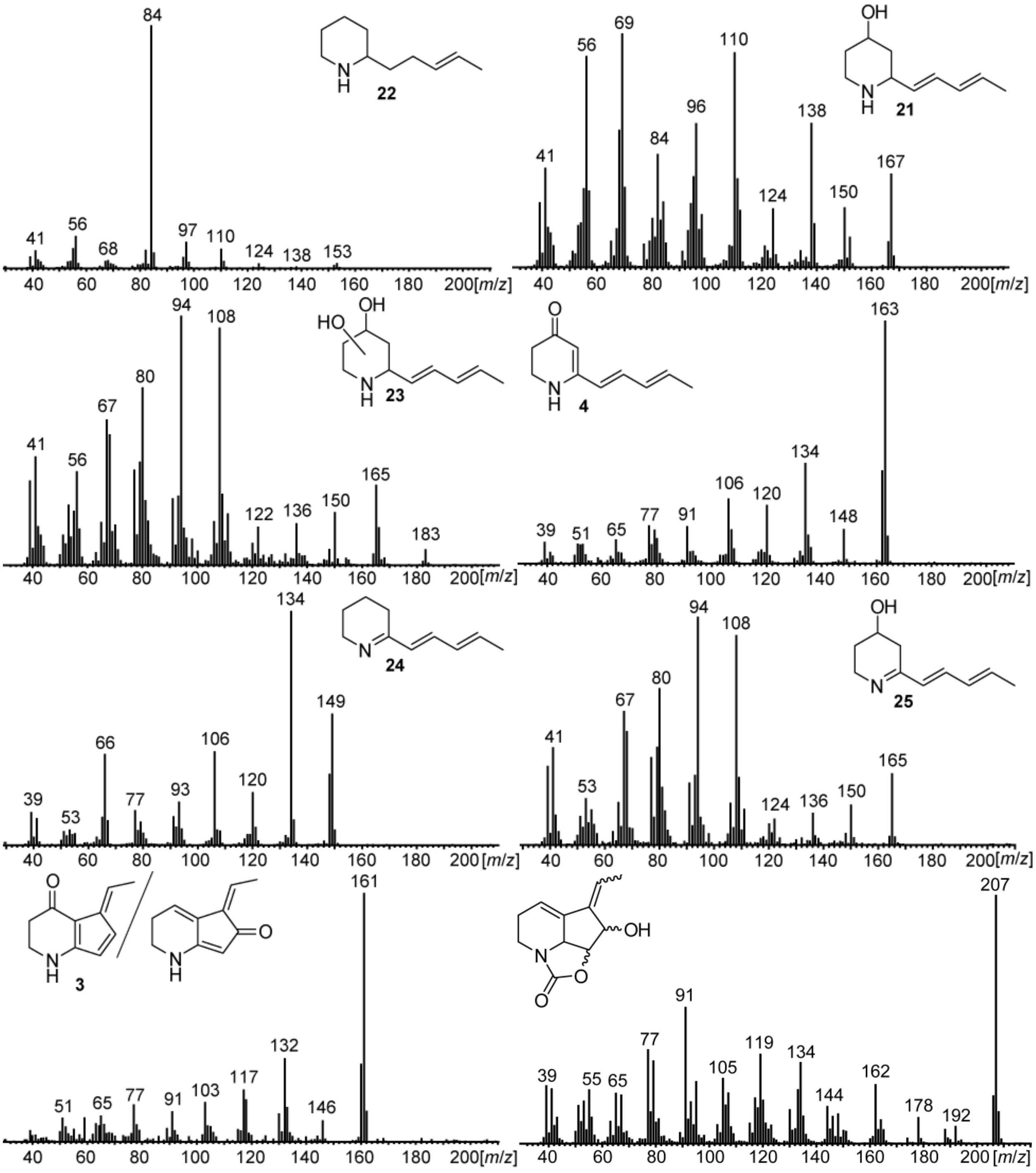

Figure S7: EI-Mass spectra of piperidine derivatives and a streptazolin stereoisomer. The structures are tentatively assigned basing on the discussion in the main text.

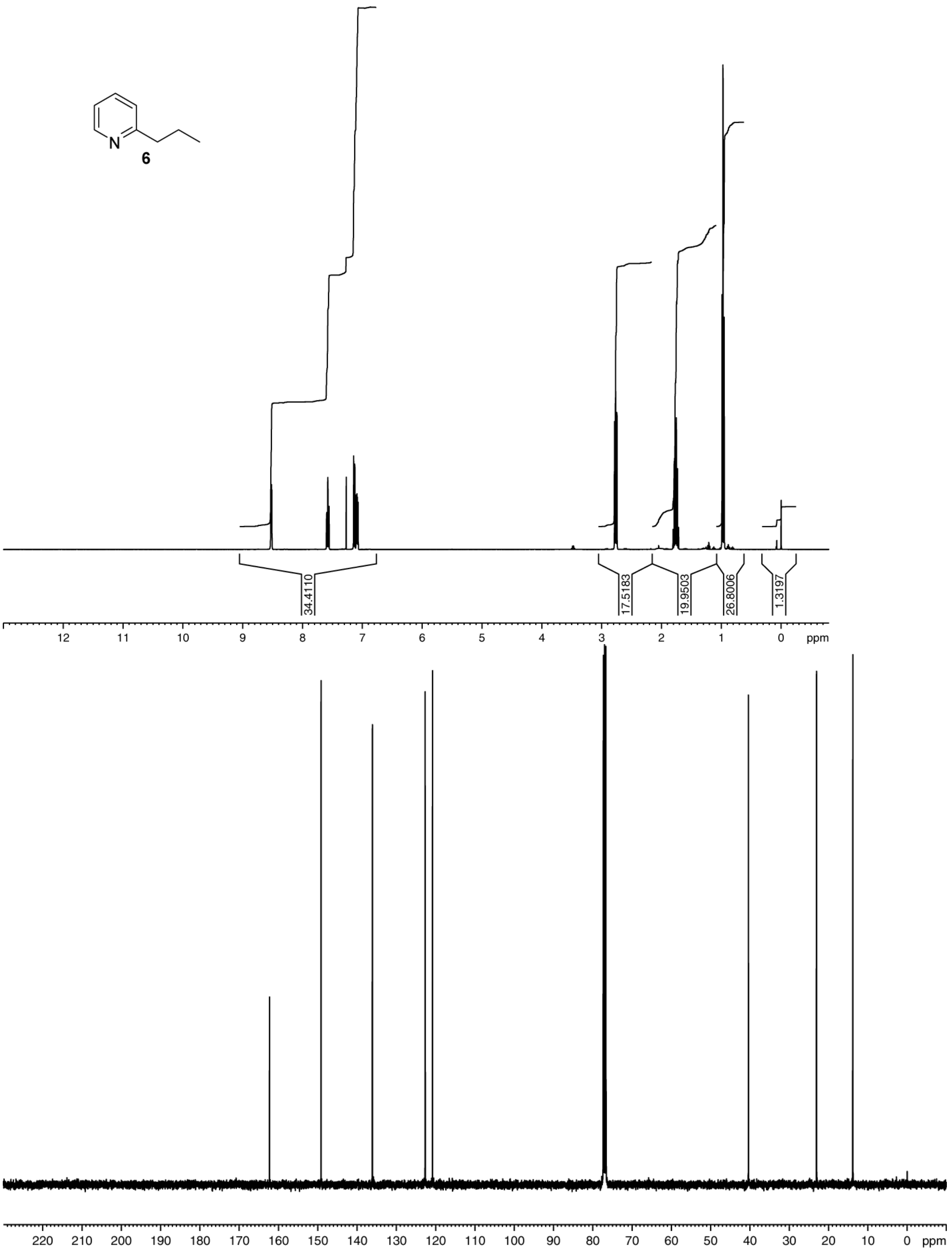

Figure S8: ^{1}H NMR ($CDCl_3$, 400 MHz) and ^{13}C NMR ($CDCl_3$, 100 MHz) spectrum of compound **6**.

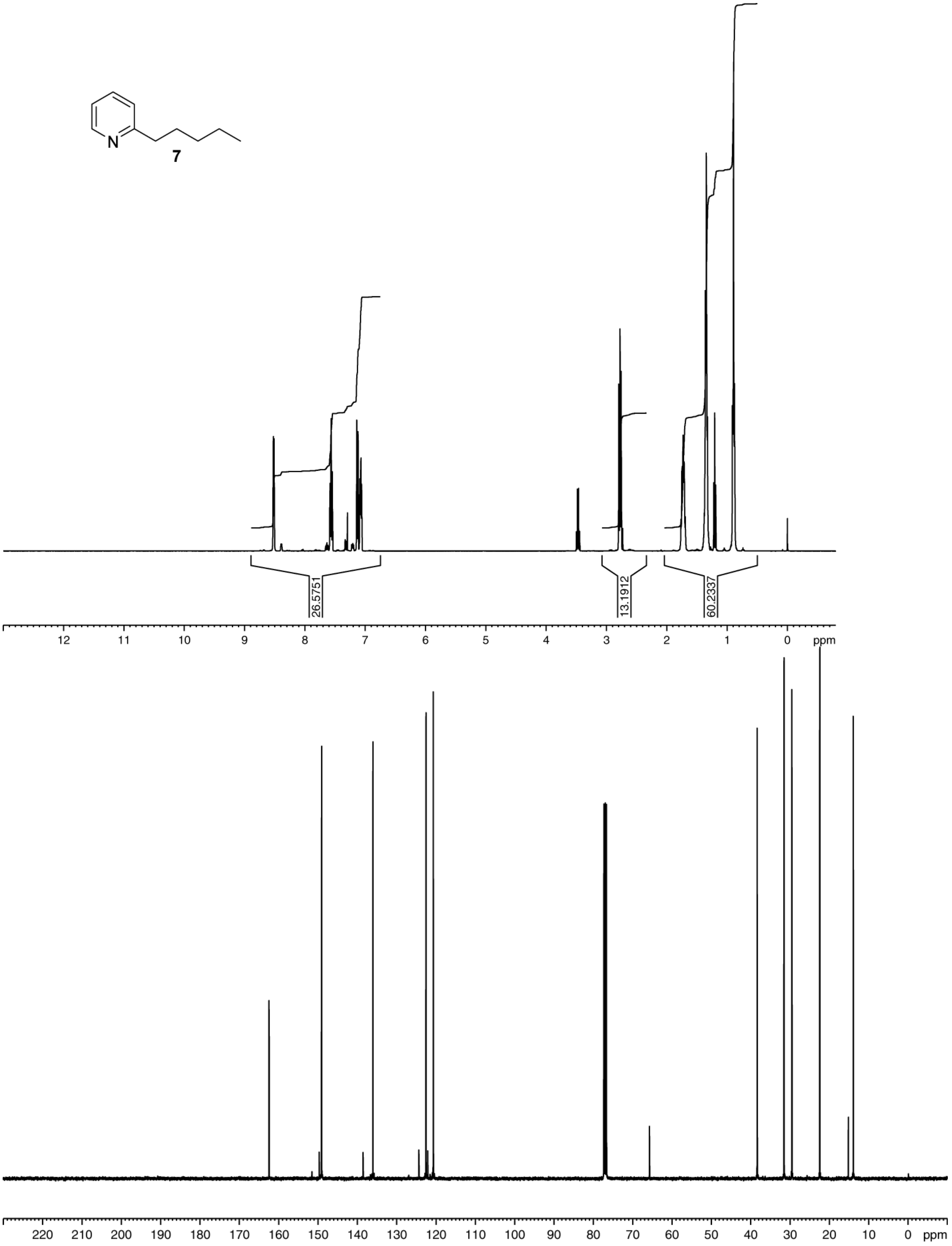

Figure S9: ^{1}H NMR ($CDCl_3$, 400 MHz) and ^{13}C NMR ($CDCl_3$, 100 MHz) spectrum of compound **7.**

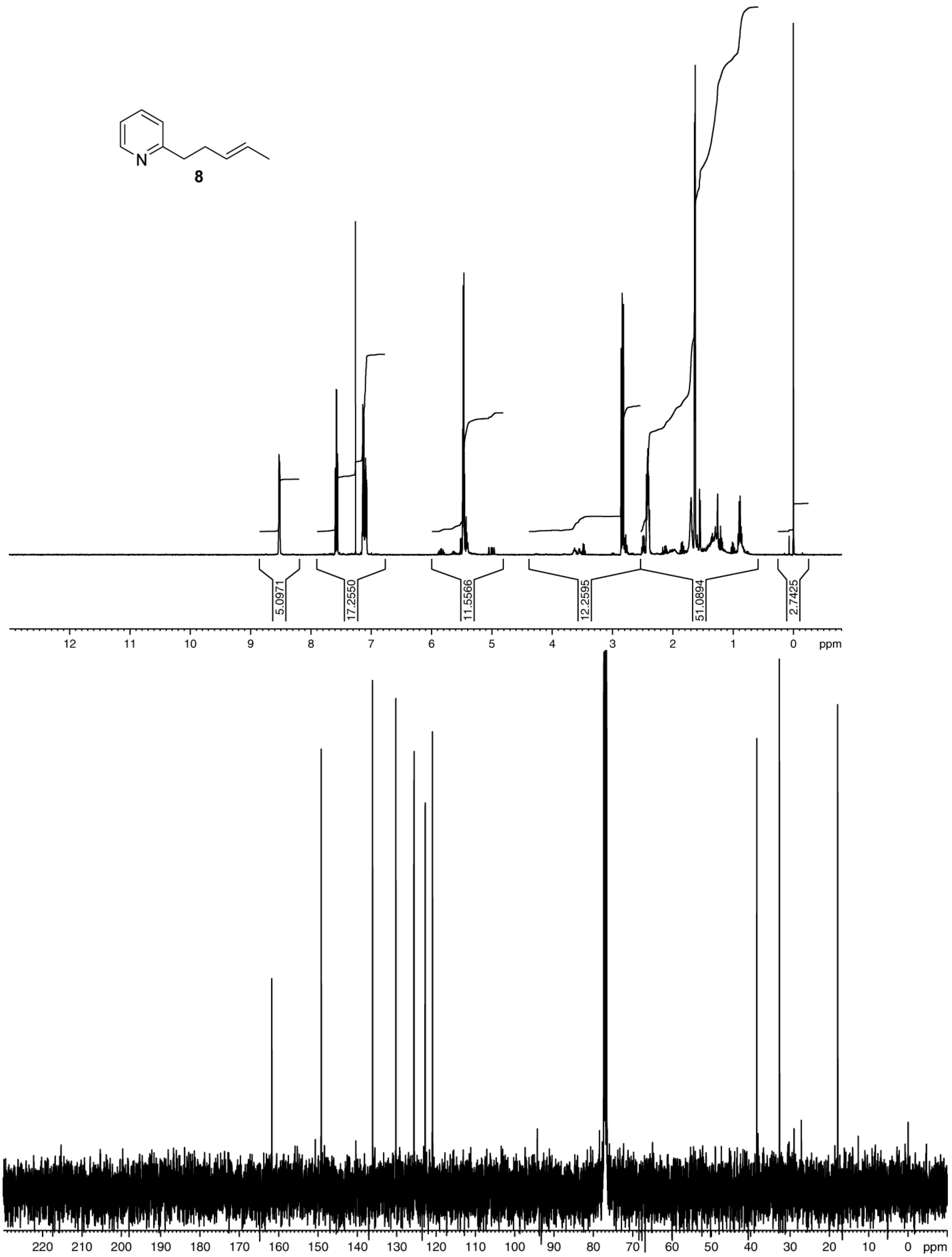

Figure S10: ^{1}H NMR ($CDCl_3$, 400 MHz) and ^{13}C NMR ($CDCl_3$, 100 MHz) spectrum of compound **8**.

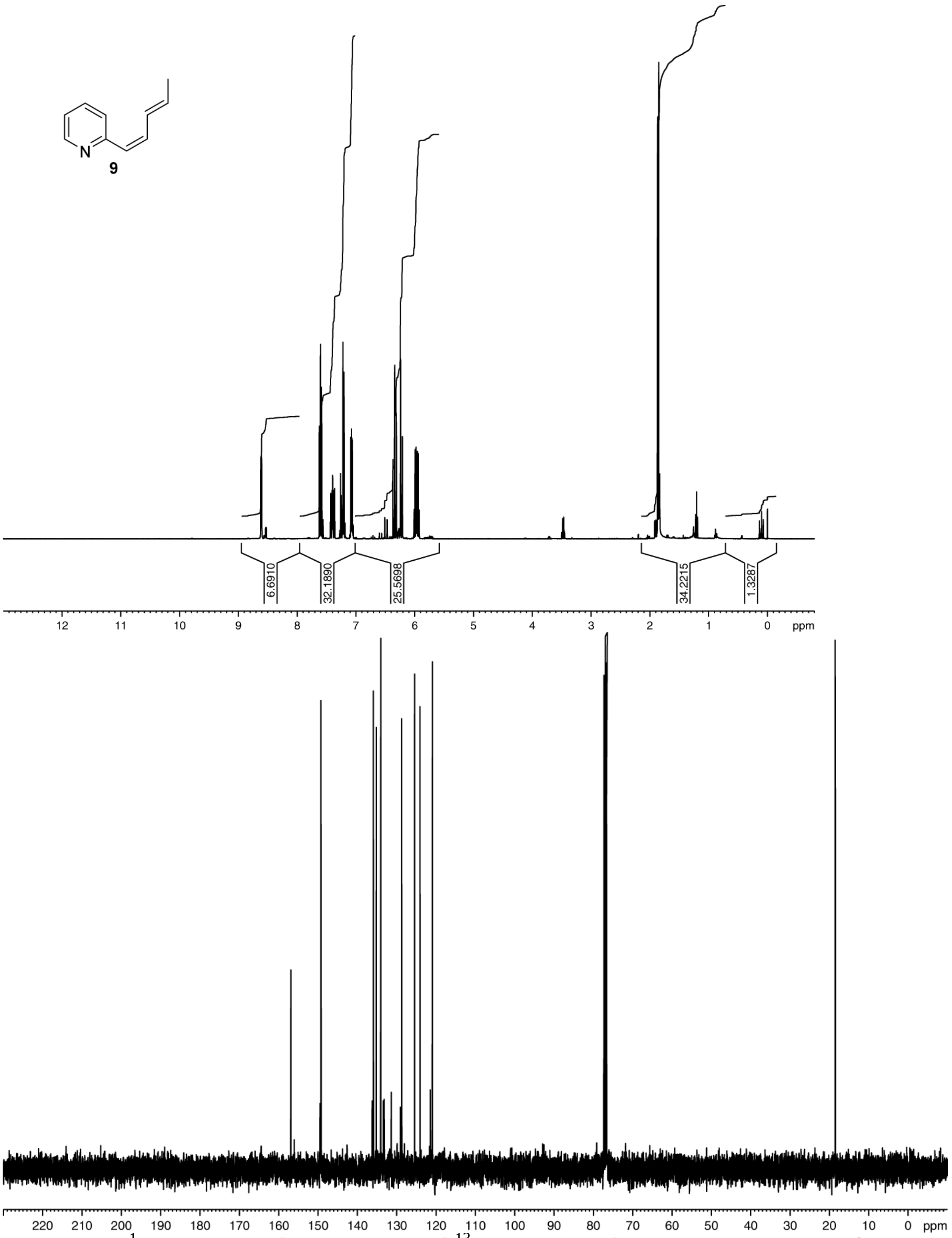

Figure S11: ^{1}H NMR ($CDCl_3$, 400 MHz) and ^{13}C NMR ($CDCl_3$, 100 MHz) spectrum of compound **9**.

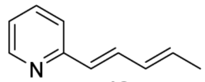

12

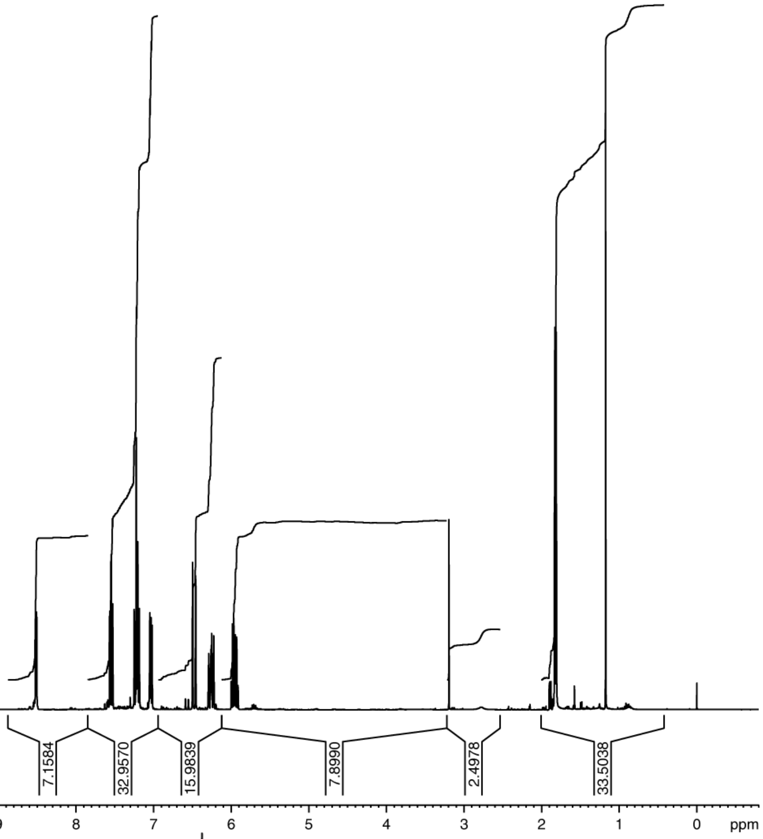

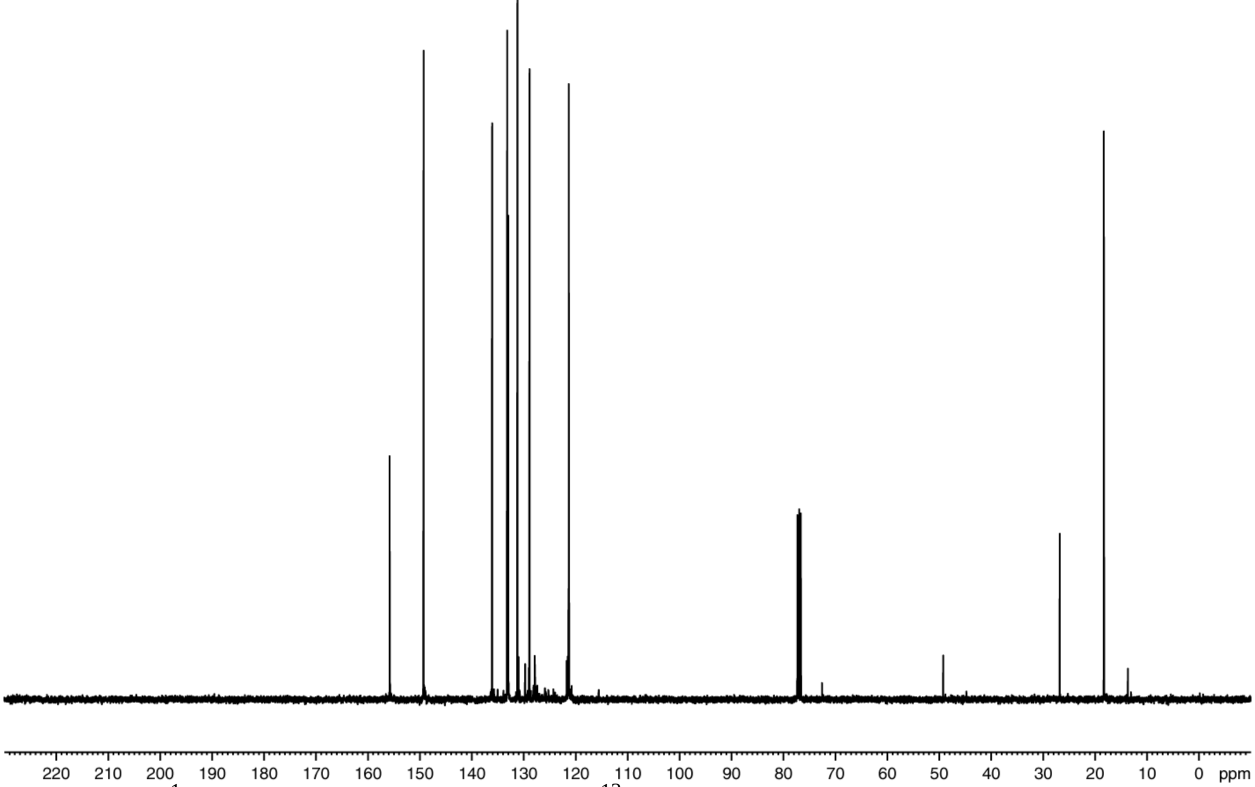

Figure S12: ^{1}H NMR ($CDCl_3$, 400 MHz) and ^{13}C NMR ($CDCl_3$, 100 MHz) spectrum of compound **12**.

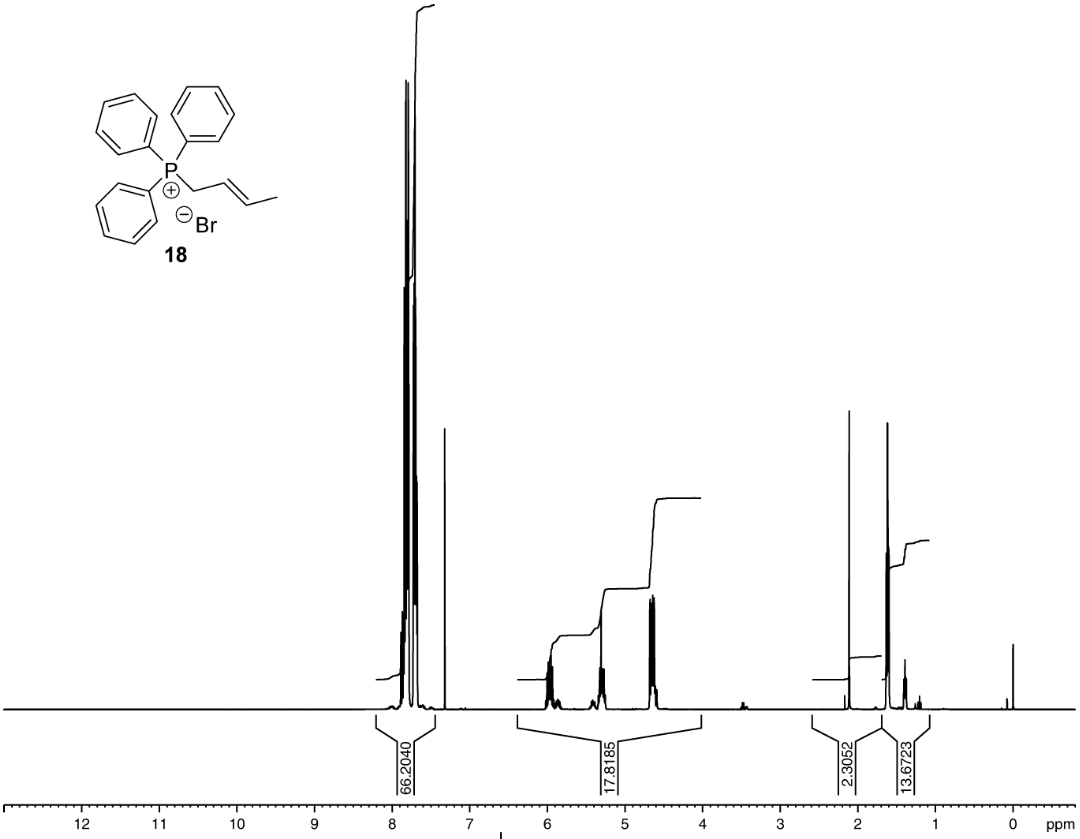
P
Br
18
66.2040
17.8185
2.3052
13.6723
12 11 10 9 8 7 6 5 4 3 2 1 0 ppm

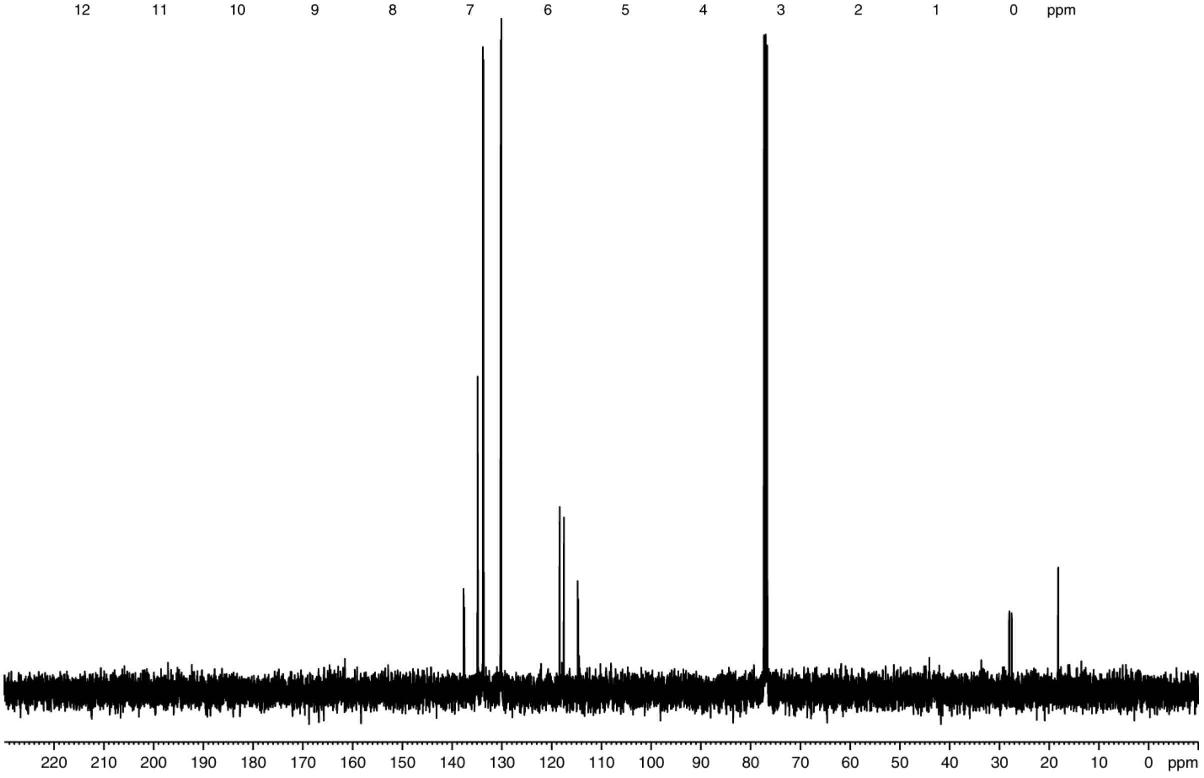
220 210 200 190 180 170 160 150 140 130 120 110 100 90 80 70 60 50 40 30 20 10 0 ppm

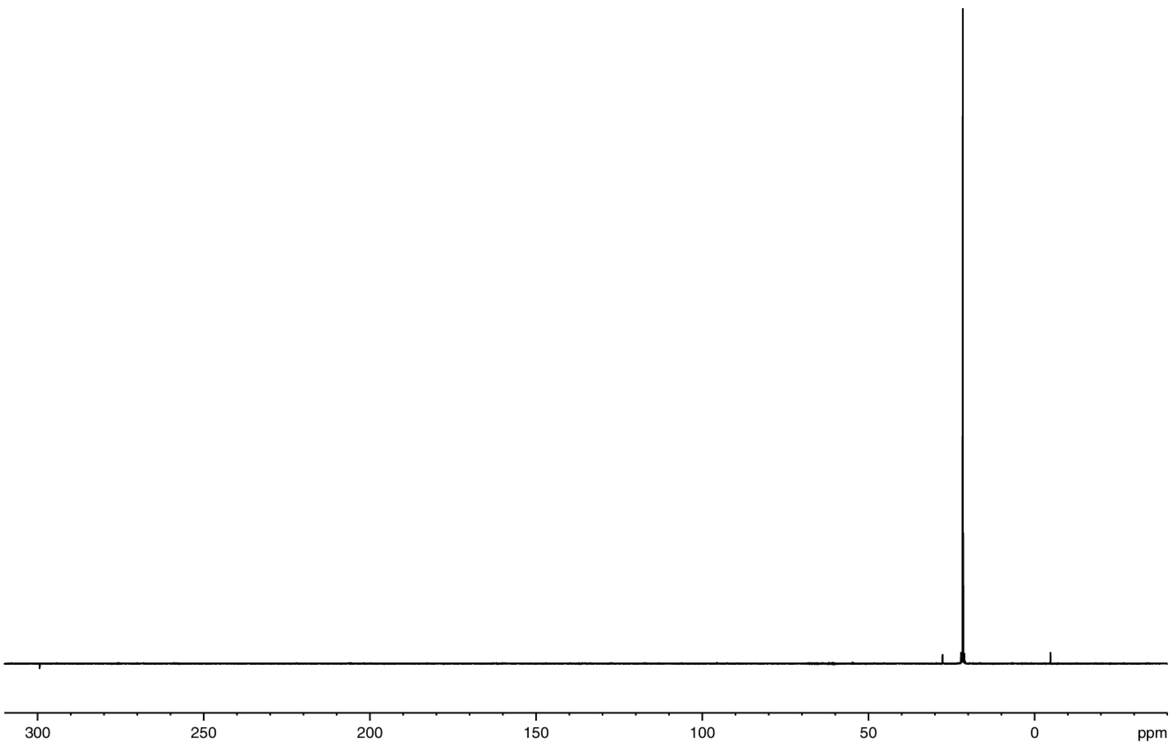

Figure S13: ^{1}H NMR ($CDCl_3$, 400 MHz), ^{13}C NMR ($CDCl_3$, 100 MHz) and ^{31}P NMR spectrum of compound **18**.
